# Nrf2 expands the intracellular pool of the chaperone AHSP in a cellular model of β-thalassemia

**DOI:** 10.1016/j.redox.2022.102239

**Published:** 2022-01-21

**Authors:** Gaijing Han, Cong Cao, Xi Yang, Guo-Wei Zhao, Xin-Jun Hu, Dong-Lin Yu, Rui-Feng Yang, Ke Yang, Ying-Ying Zhang, Wen-Tian Wang, Xiu-Zhen Liu, Peng Xu, Xue-Hui Liu, Ping Chen, Zheng Xue, De-Pei Liu, Xiang Lv

**Affiliations:** a State Key Laboratory of Medical Molecular Biology, Institute of Basic Medical Sciences, Chinese Academy of Medical Sciences & Peking Union Medical College, Beijing, 100005, PR China; b State Key Laboratory of Experimental Hematology, Institute of Hematology & Blood Diseases Hospital, Chinese Academy of Medical Sciences & Peking Union Medical College, Tianjin, 300020, PR China; c Hematology Center of Cyrus Tang Medical Institute, Soochow University, Suzhou, Jiangsu, 215123, PR China; d NHC Key Laboratory of Thalassemia Medicine, Guangxi Key Laboratory of Thalassemia Research, The First Affiliated Hospital of Guangxi Medical University, Nanning, Guangxi, 530021, PR China

**Keywords:** AHSP, α-Globin, β-thalassemia, ROS, Nrf2, MafG

## Abstract

In β-thalassemia, free α-globin chains are unstable and tend to aggregate or degrade, releasing toxic heme, porphyrins and iron, which produce reactive oxygen species (ROS). α-Hemoglobin-stabilizing protein (AHSP) is a potential modifier of β-thalassemia due to its ability to escort free α-globin and inhibit the cellular production of ROS. The influence of AHSP on the redox equilibrium raises the question of whether AHSP expression is regulated by components of ROS signaling pathways and/or canonical redox proteins. Here, we report that AHSP expression in K562 cells could be stimulated by NFE2-related factor 2 (Nrf2) and its agonist tert-butylhydroquinone (tBHQ). This tBHQ-induced increase in AHSP expression was also observed in Ter119+ mouse erythroblasts at each individual stage during terminal erythroid differentiation. We further report that the AHSP level was elevated in α-globin-overexpressing K562 cells and staged erythroblasts from β^IVS-2-654^ thalassemic mice. tBHQ treatment partially alleviated, whereas Nrf2 or AHSP knockdown exacerbated, α-globin precipitation and ROS production in fetal liver-derived thalassemic erythroid cells. MafG and Nrf2 occupancy at the MARE-1 site downstream of the AHSP transcription start site was detected in K562 cells. Finally, we show that MafG facilitated the activation of the AHSP gene in K562 cells by Nrf2. Our results demonstrate Nrf2-mediated feedback regulation of AHSP in response to excess α-globin, as occurs in β-thalassemia.

## 1 Introduction

Erythroid cells must maintain a delicate balance between α- and β-globin proteins because the individual components of the oxygen-carrying molecule hemoglobin are toxic prior to their proper assembly. Mutations that impede the synthesis of either globin chain lead to thalassemia, one of the most common human genetic disorders and a disease prevalent throughout the Mediterranean and tropical regions [1]. Unpaired α-globin in β-thalassemia is more hazardous than free β-globin in α-thalassemia because α-globin is unable to form relatively stable homotetramers as β-globin does [2]. In the clinical, patients with β-thalassemia/hemoglobin E (HbE) have higher ROS levels in red blood cells than patients with hemoglobin H (HbH) α-thalassemia [3]. Excess free α-globin chains in β-thalassemia are unstable and tend to undergo autoxidation, aggregation and degradation, resulting in the production of high amounts of ROS, free heme and iron inside erythroid cells [4]. Both heme and iron are oxidizing agents: heme promotes oxidation reactions, while iron acts as a Fenton reagent in the Haber-Weiss cycle for the generation of highly reactive hydroxyl radicals. These radicals promote extensive oxidative damage [5], resulting in a typical high-oxidative stress environment in the interior of β-thalassemic erythroid cells [6,7].

NFE2-related factor 2 (Nrf2) is a CNC-bZIP family member known for its central role in cellular oxidative defense [8,9]. Mammalian Nrf2 is normally maintained by the actin-binding protein Kelch-like ECH-associated protein-1 (Keap1) within the cytosol, where it undergoes rapid turnover via ubiquitin-mediated degradation [10,11]. When cells are stimulated by antioxidants/oxidants, modification of key cysteine residues in Keap1, and the protein kinase PKC-mediated Nrf2 phosphorylation, resulting in the release and translocation of Nrf2 into the nucleus [12]. Thereafter, Nrf2 forms a heterodimer with either a small Maf (sMaf) family protein [13] or c-Jun [14] and binds antioxidant response elements (AREs) to activate an antioxidant transcriptional program [15].

Erythroid cells contain significant amounts of α-hemoglobin-stabilizing protein (AHSP), which specifically chaperones and stabilizes the α-Hb hemichrome [16]. An *in vitro* study has shown that AHSP binds both apo-α-globin and holo-α-globin [17]. AHSP binding stabilizes both the ferrous and ferric forms of free α-Hb by facilitating histidyl coordination of heme iron, which inhibits α-Hb from generating deleterious ROS, thus preventing oxidative stress-induced precipitation [[18], [19], [20]]. In mice, AHSP ablation leads to mild hemolytic anemia and exacerbates both α- and β-thalassemia. This finding suggests that in addition to escorting excessive α-Hb, AHSP plays a role in facilitating the assembly of adult hemoglobin (HbA) [21,22]. In erythroid cells, AHSP expression is facilitated by the master erythroid transcription factors GATA binding protein 1 (GATA1) and erythroid Kruppel-like factor (EKLF) and the ubiquitous transacting factor octamer transcription factor 1 (OCT1), which directly binds the AHSP promoter to regulate its activity and/or epigenetic accessibility [16,[23], [24], [25]].

The involvement of AHSP in ROS inhibition demonstrates that AHSP acts as an antioxidant gene. This finding raises the question of whether AHSP is subject to regulation by Nrf2, particularly in thalassemic cells, where the Nrf2 pathway is strongly activated. In this study, we found that AHSP is an Nrf2 target gene whose expression in both human and mouse erythroid cells is stimulated by tBHQ and that Nrf2/MafG occupies the MARE-1 site to activate the transcription of the AHSP gene. In addition, we found that AHSP expression was significantly elevated in a β-thalassemia cellular model and in staged erythroblasts from β^IVS-2-654^ thalassemic mice and that overexpression of AHSP at least partially improved the thalassemic phenotypes of *in vitro*-cultured murine thalassemic erythroid cells. Together, these results demonstrate the Nrf2-mediated protective upregulation of AHSP in β-thalassemia.

## 2 Methods

### 2.1 Reagents and antibodies

The Nrf2-specific activator tBHQ (Sigma #112941) was dissolved in DMSO to form a 20 mM stock solution (1000 × ). *N*-Acetylcysteine (NAC) (HY–B0215) was purchased from MedChemExpress. A Dual Luciferase Reporter Assay System Kit (Pierce #20148) was purchased from Promega. An OxiSelect™ Hydrogen Peroxide/Peroxidase Assay Kit (STA-344) was purchased from Cell Biolabs. Anti-flag M2 affinity gel (Sigma A2220) and 3X flag peptide (Sigma F4799) were used to purify flag-tagged Nrf2, MafG, and MafK. CD45 microbeads (130-052-301), LS columns (130-042-401) were purchased from Miltenyi Biotec. The following polyclonal antibodies were purchased from Abcam: anti-Nrf2 (human) (EP1808Y) (ab62352), anti-MafK (ab229766) and anti-MafG (ab154318). Antibodies against AHSP (TA319485) were purchased from OriGene, hemoglobin α (sc-514378) and hemoglobin β (sc-21757) antibodies were obtained from Santa Cruz Biotechnology. An HBA2 antibody (PA5-26421) and Nrf2 (mouse) (PA5-27882) was purchased from Invitrogen. An anti-actin monoclonal antibody (ab8226, Abcam) was used as an internal control in protein level analysis. CD16/CD32 (BD, 553141), FITC-conjugated Ter-119 (BD, 557915), PE-conjugated CD44 (BD, 553134), APC-Cy7–conjugated CD45/CD11b/GR1 (BD, 557659/557657/560600) and 7-AAD (BD, 51-68981E) were used for FACS sorting of staged splenic and bone marrow Ter119+ cells.

### 2.2 Cell culture

Human chronic myelogenous leukemia (K562) cells and the murine erythroleukemia (MEL) cell line were cultured in RPMI 1640 medium (Invitrogen) containing 10% fetal bovine serum. The human embryonic kidney cell line HEK293T was maintained in Dulbecco's modified Eagle's medium (high glucose) (Invitrogen) supplemented with 10% fetal bovine serum.

### 2.3 Plasmid construction and establishment of stable cell lines

To generate instant overexpression plasmids, the coding sequences of Nrf2, MafG, MafK and HBA were cloned into the pCMV6 vector and pRSC-SFFV-E2A-GFP vector. MafK was also cloned into the pcDNA3.1(+) vector. K562 cells overexpressing HBA, Nrf2, MafG or MafK were produced by electrotransfection using an Amaxa Nucleofector Kit, according to the manufacturer's instructions or by lentiviral transfection. K562 cells transfected with an empty pCMV6 vector or pRSC-SFFV-E2A-GFP were used as controls. shRNAs were designed using the SplashRNA algorithm [26]. Two different shRNAs each were designed for the human Nrf2, mouse Nrf2 and mouse AHSP genes. ShRNAs targeting GFP or luciferase were used as negative controls. The sequences of the shRNAs are listed in Supplementary Fig. 8.

## 3 Animals

The animal experiments were approved by the animal ethics committee of the Institute of Basic Medical Sciences, Chinese Academy of Medical Sciences & Peking Union Medical College (No.ACUC-A02-2018-005). Male mice aged 6–8 weeks were used in the experiments and were maintained under specific pathogen-free conditions. β^IVS-2-654^ thalassemic mice were obtained from the Jackson Laboratory, and the mice used in this study were backcrossed to C57BL/6J mice for at least five generations. Genotyping of β^IVS-2-654^ thalassemic mice was performed via polymerase chain reaction (PCR), as previously described [27]. The primer sequences and PCR conditions are available upon request.

### 3.1 ROS detection

K562 cells overloaded with α-globin and the control cells were collected directly by centrifugation; bone marrow CD45-negative cells were isolated from 6- to 8-week-old β^IVS-2-654^ thalassemic mice and their sibling controls using CD45 microbeads, LS columns and a magnetically activated cell sorter (MACS). The ROS levels in the cells were detected following the protocol provided with an OxiSelect™ Hydrogen Peroxide/Peroxidase Assay Kit. After sonication, the cell lysates were placed in a 96-well plate and were read with a Cytation 5 fluorescence microplate reader.

### 3.2 Hematologic analysis

Peripheral blood samples were collected from β^IVS-2-654^ thalassemic mice and their sibling controls (6–8 weeks old) using EDTA-2K as an anticlotting agent. Blood smears were stained with Wright-Giemsa after air-drying. Inclusion bodies were inspected by incubating peripheral blood with an equal amount of brilliant cresyl blue dye (1 g of brilliant cresyl blue and 0.4 g of sodium citrate tribasic dehydrate dissolved in 100 ml of saline) while protected from light for 1 h at 37 °C.

### 3.3 Immunofluorescence

Cells were collected, washed, and resuspended in cold PBS. Twenty-microliter suspensions were smeared on cover slips and air-dried. Following fixation, the cells were rinsed and permeabilized with 0.1% Triton X-100 in PBS for 5 min at room temperature. After blocking for 1 h in 2% BSA/PBS, the cells were incubated overnight with anti-Nrf2 or anti-AHSP antibodies at 4 °C. Controls with no primary antibody were included to verify the specificity of the experiments. The cells were then washed with cold PBS, incubated for 1 h with a FITC-labeled anti-rabbit IgG (1:100) antibody, and stained with Hoechst for 5 min at room temperature. The signals were visualized using a confocal microscope at 200 × magnification (Zeiss Axio Scope A1 Microscope), and the Laser Sharp software program was used to record the results.

### 3.4 Cell sorting of splenic Ter119+ cells

Freshly isolated spleens from β^IVS-2-654^ thalassemic mice were pooled. To obtain single-cell suspensions, the spleens were ground and strained through a 74-μm nylon membrane in modified phosphate-buffered saline (PBS/2 mM EDTA/0.5% BSA). Ter119+ cells were isolated using a magnetically activated cell sorter (MACS) and Ter119 microbeads according to the manufacturer's instructions (Miltenyi Biotec). An aliquot of the sorted cells (10^5^) was stained with a FITC-conjugated mouse anti-Ter119 antibody (BD Biosciences) to determine the efficiency of the cell sorting process using a flow cytometric analysis on a BD Accuri C6.

### 3.5 Fluorescence-activated cell sorting of terminally differentiated mouse bone marrow erythroid cells

Fluorescence-activated sorting of murine bone marrow terminal erythroid cells was carried out following the methods in a previous publication [28]. Murine femur and tibia bone marrow cells were collected with modified phosphate-buffered saline (PBS/2 mM EDTA/0.5% BSA). Nylon membranes (74-μm) were used to filter bone debris. CD45^+^ cells were depleted through negative selection using CD45 microbeads, LS columns and MACS. The buffer was changed to PBS/0.5% BSA before antibody incubation. The cells were first incubated with rat anti–mouse CD16/CD32 for 20 min at 4 °C and then stained with FITC-conjugated rat anti–mouse Ter-119, PE-conjugated rat anti–mouse CD44, and APC-Cy7–conjugated rat anti-mouse CD45/CD11b/GR1 for 30 min at 4 °C. After washing with PBS/0.5% BSA, the cells were resuspended in RPMI 1640 culture medium and incubated with 7-AAD for 10 min at 4 °C. Finally, the cells were diluted in RPMI 1640 culture medium and sorted using BD FACS Aria SORP flow cytometry.

### 3.6 *In vitro* erythroid differentiation of mouse fetal liver Ter119^-^ cells

Fetal liver cells were isolated from E14.5 mouse embryos, the total cells were labeled with anti-Ter119 MicroBeads (Miltenyi Biotec, 130-049-901), and Ter119-negative cells were purified through LD Columns (Miltenyi Biotec, 130-042-901) as per the manufacturer's instructions. The purified cells were cultured in erythroid-proliferation medium (EPM) (Iscove's modified Dulbecco's medium (IMDM) containing 25% FBS, 1% BSA, 10 μg/ml recombinant human insulin, 2 mM l-glutamine, and 10^−4^ M β-mercaptoethanol, 10^−4^ M Dexamethasone, 20 ng/ml IL-3, 0.4% Cholesterol, 40 ng/ml IGF-1, 200 μg/ml holo-transferrin, 10 U/ml Epo and 100 ng/ml SCF). Four days later, the medium was replaced with erythroid differentiation medium (EDM) (IMDM containing 5% FBS, 100 ng/ml SCF, 3 U/ml Epo, 500 μg/ml holo-transferrin, 1-Thioglycerol, 10% Serum Replacement and 10% PFHM-II), and the cells were cultured for 3–4 days. tBHQ treatment was administered during the *in vitro* erythroid differentiation stage. Isolated mouse fetal liver Ter119-negative cells were proliferated in EPM for 4 days before transfer to EDM with DMSO or 20 μM tBHQ for another 4 days of differentiation. Retroviruses expressing shRNA were used to infect EPM-cultured mouse fetal liver Ter119-negative cells for 24 h before differentiation in EDM for 4 days. The cells were labeled with antibodies against Ter119-PE (BD553673) and CD71-APC (eBioscience 17-0711-82) to assess the status of differentiation.

### 3.7 RNA extraction and real-time RT-PCR

Total RNA was extracted from differentially treated K562 cells and mouse Ter119+ cells using TRIzol Reagent or from FACS-isolated terminal differentiation erythroid cells using a MicroElute Total RNA Kit (OMIGA, R6831-01) according to the manufacturer's instructions. First-strand cDNA was synthesized using 3 μg of RNA as a template, M-MLV reverse transcriptase, and random primers. Real-time RT-PCR amplifications were performed using an iQTM5 Real-Time PCR Detection System (Bio-Rad), SYBR Green PCR Mix, and the forward/reverse primer pairs shown in Supplementary Table 1. The qPCR amplification conditions included 95 °C for 10 min, followed by 39 cycles of amplification at 95 °C for 10 s, 60 °C for 30 s, 72 °C for 30 s, and 72 °C for 5 min as the final elongation step. AHSP expression was quantified by normalizing the data against the β-actin control values using the delta Ct method.

### 3.8 Western blotting analysis

Pellets of differentially treated K562 cells or mouse splenic Ter119+ cells were collected and lysed in RIPA buffer (20 mM Tris-HCl [pH 7.4] 5 mM EDTA, 1 mM β-mercaptoethanol, 10% glycerol, 1% Triton X-100, 150 mM NaCl, 0.1 mM PMSF and protease inhibitor cocktail). Following incubation on ice with occasional rotation, the lysate was centrifuged at 13,000 rpm at 4 °C for 15 min, and the protein concentration in the supernatant was quantified using a Pierce BCA protein assay kit. Depending on the protein to be detected, approximately 10–50 μg of total extract proteins (the same protein load was used in each gel) were loaded into each lane of a 12% SDS-PAGE gel. The PVDF membranes were incubated overnight with the appropriate primary antibodies and were then probed with the corresponding secondary antibodies for 1–2 h at room temperature. The protein bands were visualized using an ECL chemiluminescence kit.

### 3.9 Chromatin immunoprecipitation

Chromatin immunoprecipitation (ChIP) analysis of control or α-globin-overexpressing K562 cells was performed as previously described, with minor modifications [29]. Equivalent amounts of chromatin were sonicated by pulsing eight times at 8 W for 10 s each time at 1 min intervals. Next, the samples were centrifuged at 14,000 rpm (20,817×*g*) for 15 min to obtain cleared cell lysates, which were immunoprecipitated with 2–8 μl (4 μg) of one of the following primary antibodies: anti-Nrf2, anti-MafG or anti-MafK. The control consisted of 10 μl (4 μg) of normal rabbit IgG. The precipitated DNA was dissolved in 100 μl ddH_2_O and was analyzed by real-time qPCR using an iQTM5 Real-Time PCR Detection System and SYBR Green PCR Mix. The PCR primers used to amplify the MARE-1 site from the immunoprecipitated chromatin were: forward 5′-TCCATTTCAAAGGACTGGCC-3′ and reverse 5′-CACAGCACCAACTCACAT-3’ (product size: 154 bp). The primers for the negative control (NC) site (hg 38, chr16:31540577–31540727) were forward 5′-CCCAGATCTTTCTTTTCGGGC-3′ and reverse 5′- GGGAAACTGCCAAGCCTCTA-3’ (product size: 151 bp). qPCR amplification conditions included 95 °C for 10 min, followed by 39 cycles of amplification at 95 °C for 10 s, 60 °C for 30 s, 72 °C for 30 s and 72 °C for 5 min as the final elongation step. The data from the immunoprecipitated DNA were normalized against the values from the input genomic DNA. The y-axes of the ChIP graphs indicate the enrichment relative to the input.

### 3.10 Reporter plasmid construction and luciferase reporter assay

The pGL3-promoter-MARE-1-WT reporter plasmid was constructed by PCR amplification of an ∼0.5 kb region (+52 to +552 bp) downstream of the AHSP transcription start site and insertion of the fragment into the *Nhe*I/*Xho*I restriction sites of the pGL3-promoter vector. The pGL3-promoter-MARE-1-Mut reporter plasmid was constructed with a Fast mutagenesis system (TransGen Biotech, FM111-02). HEK293T cells at 50% confluency were transfected with firefly reporter plasmids (0.9 μg), Renilla luciferase transfection efficiency control (pRL-CMV, 5 ng), and the appropriate expression or empty vector (0.9 μg). The transfections were performed in 24-well plates using VigoFect according to the manufacturer's instructions, and the cells were harvested after 24 h. Luciferase activity was measured using a Dual Luciferase Reporter Assay System Kit. The firefly values were normalized to the Renilla values, and the activity values determined from at least three separate transfection experiments are presented as the relative luciferase values.

### 3.11 Electrophoretic mobility shift assay (EMSA)

Flag-tagged Nrf2, MafG or MafK was overexpressed in HEK293T cells and purified with an anti-flag M2 affinity gel and a 3X flag peptide. An EMSA was performed using a LightShift Chemiluminescent EMSA Kit (Pierce #20148) according to the manufacturer's instructions. Each 20 μl reaction contained 8 μg of purified protein and 20 nM labeled and annealed probes (forward: biotin-5′-TTGAAGGAGTTCAGCGTTCTGCTGAATCAGCAGGTGAGTCCAAGC-3′, reverse: biotin-5′-GCTTGGACTCACCTGCTGATTCAGCAGAACGCTGAACTCCTTCAA-3′). For the competition experiments, the purified protein was incubated with a 100-fold excess of unlabeled probe for 15 min at room temperature prior to the addition of the labeled probes. The total reaction mixtures were incubated with the labeled probe for an additional 30 min and were then separated on nondenaturing 6% polyacrylamide gels in Tris-borate-EDTA (TBE) buffer. The DNA was transferred to nylon membranes and cross-linked with UV light (150 mJ/cm^2^). The membranes were then blocked, conjugated, washed, equilibrated, and exposed to X-ray film.

### 3.12 Statistical analysis

The data are expressed as the mean ± standard deviation. Analysis of variance followed by the Student-Newman-Keuls multiple comparisons test was used to identify significant differences between the groups. *p* values of less than 0.05 were considered to indicate significance and are indicated with a single asterisk (*), *p* values of less than 0.01 are indicated with double asterisks (**), and *p* values of less than 0.001 are indicated with triple asterisks (***).

## 4 Results

### 4.1 ROS production in β-thalassemia models activates the Nrf2 antioxidant response signaling pathway

**Fig. 1:**
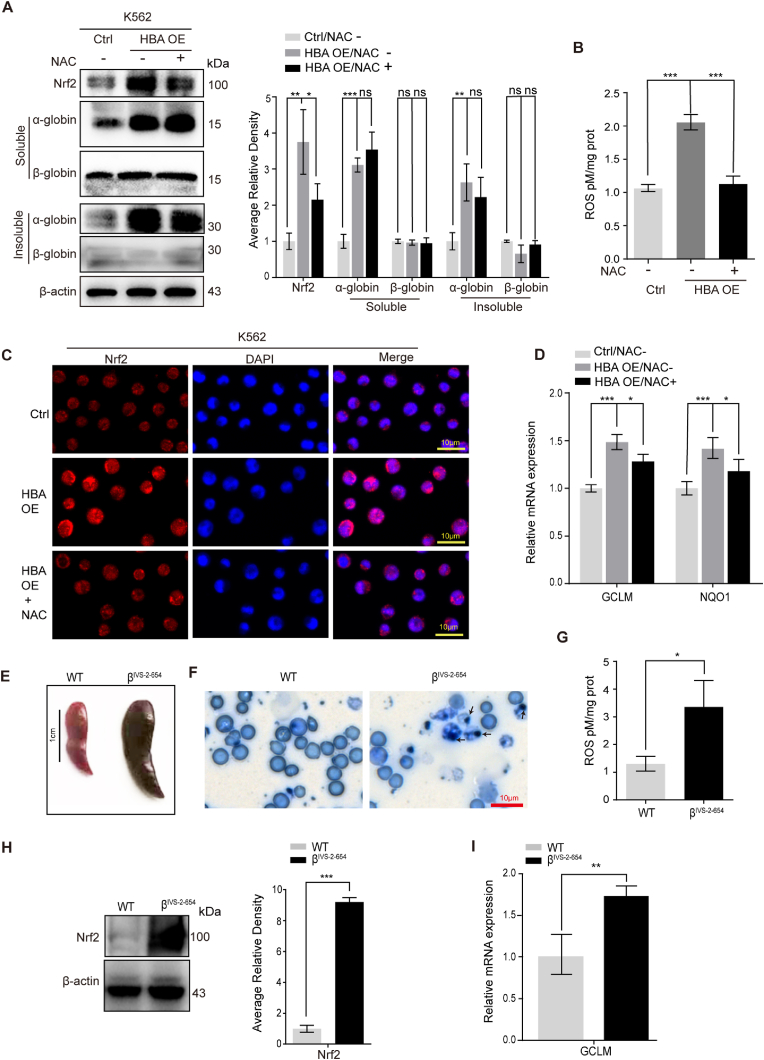
ROS activate Nrf2 in β-thalassemia models. (A) Representative Western blot analysis of Nrf2, soluble and insoluble α-globin and β-globin in control, α-globin-overexpressing and NAC-treated α-globin-overexpressing K562 cells. Nrf2, soluble and insoluble α-globin and β-globin levels were assessed by densitometric quantification and normalized to β-actin levels (the data are presented as the mean ± SD; ns, not significant; *, *P* < 0.05; **, *P* < 0.01; ***, *P* < 0.005; *n* = 4 *replicates*). (B) ROS detection assay in control, α-globin-overexpressing K562 cells and NAC-treated α-globin-overexpressing K562 cells (the data are presented as the mean ± SD; ***, *P* < 0.005; *n* = 3 *replicates*). (C) Immunofluorescence analysis of Nrf2 cellular localization in control, α-globin-overexpressing K562 cells and NAC-treated α-globin-overexpressing K562 cells. (D) Real-time PCR analysis of GCLM and NQO1 expression in control, α-globin-overexpressing K562 cells and NAC-treated α-globin-overexpressing K562 cells (the data are presented as the mean ± SD; *, *P* < 0.05; ***, *P* < 0.005; *n* = 3 *replicates*). (E) Spleens from WT and β^IVS-2-654^ thalassemic mice. (F) Inclusion body staining (indicated by arrows) of peripheral blood smears from WT and β^IVS-2-654^ thalassemic mice. (G) ROS detection assay in Ter119+ cells from WT and β^IVS-2-654^ thalassemic mice (the data are presented as the mean ± SD; *, *P* < 0.05; WT, *n* = 3 *mice;* β^IVS-2-654^, *n* = 3 *mice*). (H) Western blot analysis of the expression of Nrf2 in fetal liver derived erythroid cells from WT or β^IVS-2-654^ thalassemic mice (E14.5). Nrf2 levels were assessed by densitometric quantification and normalized to β-actin levels. (the data are presented as the mean ± SD; ***, *P* < 0.005, WT, *n* = 3 *fetal livers;* β^IVS-2-654^, *n* = 3 *fetal livers*). (I) Real-time PCR analysis of GCLM expression in bone marrow Ter119+ cells from WT and β^IVS-2-654^ thalassemic mice (the data are presented as the mean ± SD; **, *P* < 0.01; WT, *n* = 3 *mice;* β^IVS-2-654^, *n* = 3 *mice*).

Free globin is a potent oxidant that catalyzes the production of ROS, which is the most important event in the pathophysiology of thalassemia. First, we produced a cellular model of β-thalassemia by stably overexpressing α-globin in K562 cells. We used K562 cells transfected with the empty pCMV6 vector as negative controls. α-Globin overexpression in K562 cells disrupted the balance between α- and β-globin, induced the accumulation of both soluble and insoluble α-globin (Fig. 1A), and caused α-globin aggregation (Supplementary Fig. 1A). We found no obvious change in β-globin expression but observed activation of Nrf2 upon the exogenous α-globin overexpression. As expected, increased ROS levels were detected by hydrogen peroxide assay (Fig. 1B), and immunofluorescence analysis revealed the nuclear translocation of Nrf2 in the α-globin overloaded model cells (Fig. 1C). Furthermore, real-time PCR confirmed activation of the known Nrf2 target gene NAD(P)H quinone dehydrogenase 1 (NQO1) and glutamate-cysteine ligase modifier subunit (GCLM), which served as biomarkers for activation of Nrf2 in K562 cells (Fig. 1D). In addition, inhibiting ROS production with 10 mM *N*-Acetylcysteine (NAC) treatment for 24 h (Fig. 1B) partially reversed the Nrf2 activation, Nrf2 nuclear translocation and Nrf2 target gene upregulation (Fig. 1A–D).

We further examined ROS levels and Nrf2 activity in a murine model of β-thalassemia. As shown in Supplementary Fig. 1B, heterozygous β^IVS-2-654^ mice carried a splicing-deficient human β-globin allele (*hbb*^*th−4*^), and the genotype of β^IVS-2-654^ mice was identified by PCR amplification (Supplementary Fig. 1C). These mice presented β-thalassemia intermedia syndrome and obvious splenomegaly, indicating the presence of extramedullary erythropoiesis, unlike their wild-type (WT) littermates (Fig. 1E). Comparison of peripheral blood smears showed elevated poikilocytosis (Supplementary Fig. 1D) and apparent inclusion bodies (Fig. 1F) indicating α-globin precipitation in β^IVS-2-654^ erythrocytes. The accumulation of free α-globin increased endogenous ROS levels (Fig. 1G) and upregulated the Nrf2 antioxidant signaling pathway, as evidenced by increased expression of Nrf2 (Fig. 1H) and activation of the canonical Nrf2 target gene GCLM (Fig. 1I) in β^IVS-2-654^ erythroid cells.

### 4.2 Nrf2 and its agonist tBHQ upregulate AHSP in K562 cells

**Fig. 2:**
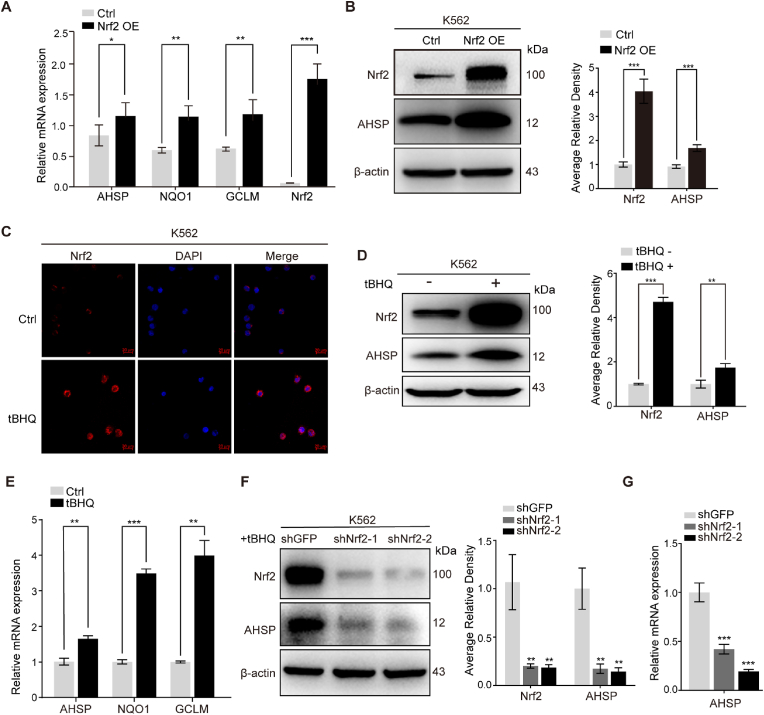
Nrf2 enhances AHSP expression in K562 cells. (A) Real-time PCR analysis of the expression of AHSP, NQO1 and GCLM in Nrf2-overexpressing and control K562 cells (the data are presented as the mean ± SD; *, *P* < 0.05; **, *P* < 0.01; ***, *P* < 0.005; *n* = 3 *replicates*). (B) Representative Western blot analysis of AHSP expression in Nrf2-overexpressing and control K562 cells. AHSP and Nrf2 levels were assessed by densitometric quantification and normalized to β-actin levels (the data are presented as the mean ± SD; ***, *P* < 0.005; *n* = 4 *replicates*). (C) Immunofluorescence analysis of Nrf2 nuclear translocation in tBHQ-treated K562 cells. (D) Representative Western blot analysis of AHSP and Nrf2 expression in tBHQ-treated and control K562 cells. AHSP and Nrf2 levels were assessed by densitometric quantification and normalized to β-actin levels (the data are presented as the mean ± SD; **, *P* < 0.01; ***, *P* < 0.005; *n* = 5 *replicates*). (E) Real-time PCR analysis of AHSP, NQO1 and GCLM expression in tBHQ-treated and control K562 cells (the data are presented as the mean ± SD; **, *P* < 0.01; ***, *P* < 0.005; *n* = 3 *replicates*). (F) Representative Western blot analysis of Nrf2 and AHSP expression in tBHQ-treated control and Nrf2-knockdown K562 cells. AHSP and Nrf2 levels were assessed by densitometric quantification and normalized to β-actin levels (the data are presented as the mean ± SD; **, *P* < 0.01; *n* = 4 *replicates*). (G) Real-time PCR analyses of AHSP expression in tBHQ-treated K562-shNrf2 and shGFP control cells (the data are presented as the mean ± SD; ***, *P* < 0.005; *n* = 3 *replicates*).

To determine whether Nrf2 regulates AHSP expression, K562 cells were stably transfected with Nrf2-overexpression (pRSC-SFFV-E2A-GFP-Nrf2) and control (pRSC-SFFV-E2A-GFP) plasmids. The overexpression of Nrf2 in K562-Nrf2 cells was confirmed by comparing Nrf2 levels and the expression of Nrf2 target genes (NQO1 and GCLM) against the basal levels in the control cells. Both real-time PCR and Western blotting showed that AHSP expression was upregulated following Nrf2 overexpression (Fig. 2A and B). tBHQ has been well characterized as a specific activator of Nrf2. Therefore, we treated K562 cells with tBHQ (20 μM for 6 h) to determine its effects on AHSP expression. Immunofluorescence analyses showed that the Nrf2 protein accumulated within the nucleus following tBHQ stimulation (Fig. 2C). AHSP expression was also increased by tBHQ treatment, as detected by both Western blotting and real-time PCR (Fig. 2D and E). Nrf2 activation and AHSP upregulation were also observed in K562 cells treated with another Nrf2 agonist, DEM (100 μM for 1–24 h) (Supplementary Fig. 2). In contrast, the upregulation of AHSP induced by tBHQ was largely impaired in Nrf2-knockdown K562 cells compared with control (K562-shGFP) cells (Fig. 2F and G), indicating that AHSP is a target gene downstream of tBHQ/Nrf2 signaling.

### 4.3 tBHQ induces AHSP overexpression in Ter119+ mouse cells

**Fig. 3:**
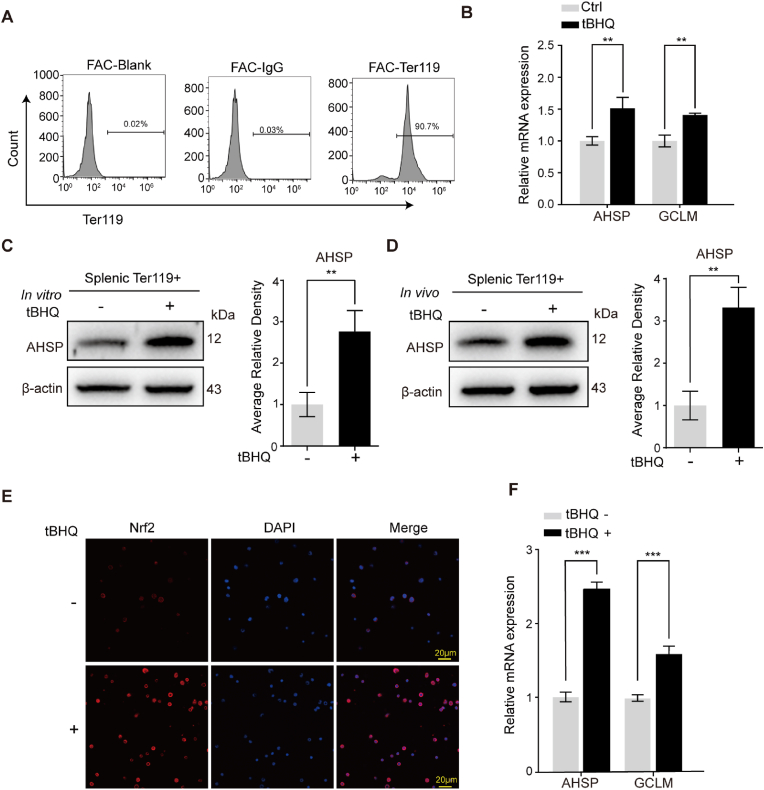
The Nrf2 agonist tBHQ stimulates AHSP expression in wild-type mouse Ter119+ cells. (A) FACS analysis of the magnetic cell sorting efficiency and purity of Ter119+ cells isolated from mouse spleens. (B) Real-time PCR analysis of AHSP and GCLM expression levels in splenic Ter119+ cells from wild-type mice with or without *in vitro* tBHQ treatment for 6 h (the data are presented as the mean ± SD; **, *P* < 0.01; *n* = 3 *replicates*). (C) Representative Western blot analysis of AHSP expression in splenic Ter119+ cells from wild-type mice with or without *in vitro* tBHQ treatment for 6 h. AHSP levels were assessed by densitometric quantification and normalized to β-actin levels (the data are presented as the mean ± SD; **, *P* < 0.01; *n* = 3 *replicates*). (D) Representative Western blot analysis of AHSP expression in splenic Ter119+ cells from wild-type mice with or without tBHQ treatment via tail vein injection. AHSP levels were assessed by densitometric quantification and normalized to β-actin levels (the data are presented as the mean ± SD; **, *P* < 0.01; *n* = 3 *replicates*). (E-F) Immunofluorescence analysis of Nrf2 nuclear translocation (E) and real-time PCR analysis of AHSP and GCLM expression (F) in bone marrow Ter119+ cells from wild-type mice with or without tBHQ treatment via intraperitoneal injection (the data are presented as the mean ± SD; ***, *P* < 0.005; *n* = 3 *replicates*).

Next, we determined the effects of tBHQ/Nrf2 on AHSP expression in mouse splenic erythroid cells. Ter119+ cells were isolated from mouse spleens using magnetic beads, and the purity of the magnetically isolated cells was confirmed using fluorescence-activated cell sorting (FACS) (Fig. 3A). In the isolated Ter119+ cells, AHSP expression was obviously upregulated after treatment *in vitro* with 20 μM tBHQ for 6 h (Fig. 3B and C), and GCLM upregulation served as a biomarker for Nrf2 activation in mouse Ter119+ cells. In addition, Ter119+ cells were isolated from mice treated with tBHQ (50 μg/g of body weight) via three intravenous injections into the tail vein (Fig. 3D) or treated with tBHQ (50 μg/g of body weight) via three intraperitoneal injections with 8 h between each injection (Fig. 3E and F). As shown in Fig. 3D, E and F, tBHQ delivered by both methods upregulated AHSP expression in mouse Ter119+ cells.

### 4.4 tBHQ upregulates AHSP during erythroid differentiation

**Fig. 4:**
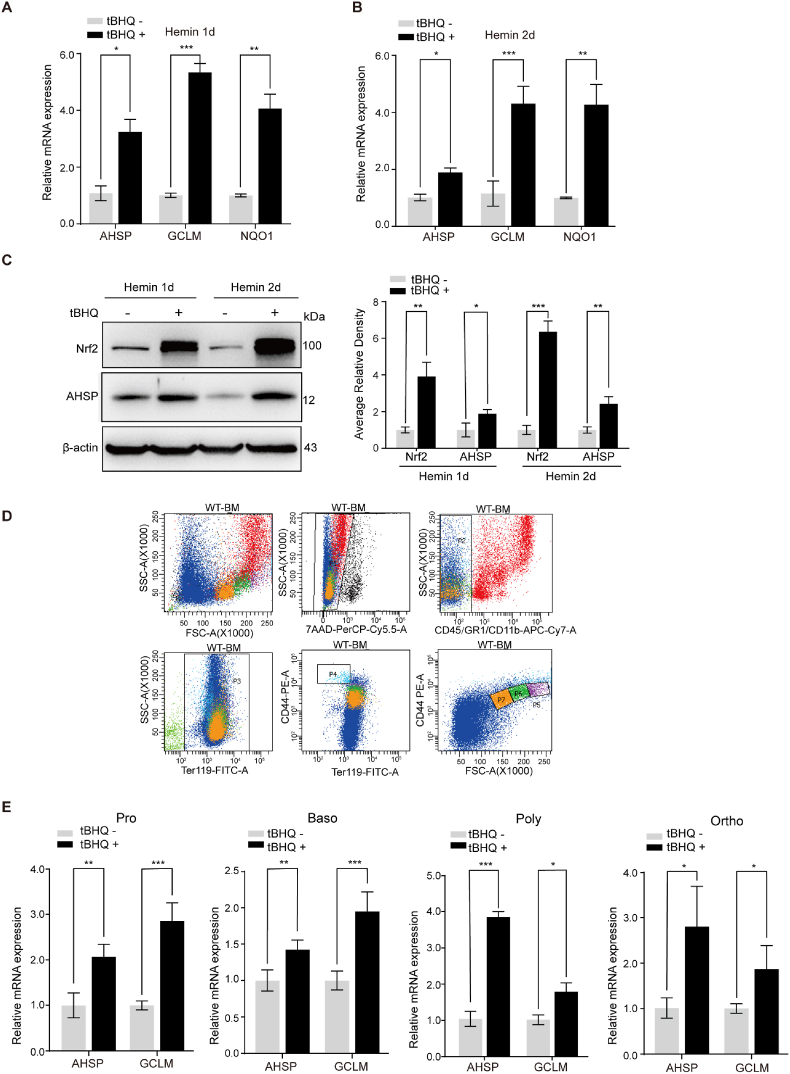
tBHQ stimulates AHSP expression during erythroid differentiation. (A-B) Real-time PCR analysis of AHSP, GCLM and NQO1 expression in K562 cells exposed to 10 μM hemin for 1 day (A) or 2 days (B) with or without tBHQ treatment for 6 h (the data are presented as the mean ± SD; *, *P* < 0.05; **, *P* < 0.01; ***, *P* < 0.005; *n* = 3 *replicates*). (C) Representative Western blot analysis of Nrf2 and AHSP expression in hemin-exposed K562 cells with or without tBHQ treatment for 6 h. AHSP and Nrf2 levels were assessed by densitometric quantification and normalized to β-actin levels (the data are presented as the mean ± SD; *, *P* < 0.05; **, *P* < 0.01; ***, *P* < 0.005; *n* = 3 *replicates*). (D) FACS isolation of staged erythroblasts from the bone marrow cells of wild-type mice. Regions P4 to P7 are proerythroblasts (Pro), basophilic erythroblasts (Baso), polychromatic erythroblasts (Poly) and orthochromatic erythroblasts (Ortho), respectively. (E) Real-time PCR analysis of AHSP and GCLM expression in wild-type mouse bone marrow proerythroblasts (Pro), basophilic erythroblasts (Baso), polychromatic erythroblasts (Poly) and orthochromatic erythroblasts (Ortho) with or without tBHQ treatment for 6 h (the data are presented as the mean ± SD; *, *P* < 0.05; **, *P* < 0.01; ***, *P* < 0.005; *n* = 3 *replicates*).

AHSP is a molecular chaperone of free α-globin. Among fetal tissues, AHSP was detected highly expressed in the fetal liver (Supplementary Fig. 3A), and its expression was upregulated along with that of α-globin during erythroid differentiation simulated by hemin in K562 cells (Supplementary Fig. 3B) and stimulated by DMSO in murine erythroleukemia (MEL) cells (Supplementary Fig. 3C). To detect AHSP expression in response to tBHQ during erythroid differentiation, we exposed K562 cells to hemin for 1 day or 2 days and treated them with 20 μM tBHQ for 6 h before harvest. As shown in Fig. 4A and B, the expression of AHSP and the known Nrf2 target genes GCLM and NQO1 was upregulated upon tBHQ treatment in K562 cells exposed to hemin for 1 day or 2 days. Western blot analysis also showed Nrf2 activation and increased AHSP expression with tBHQ stimulation in K562 cells during hemin-induced erythroid differentiation (Fig. 4C). To confirm that tBHQ increased AHSP expression during *in vivo* erythroid differentiation, we sorted Ter119+ mouse bone marrow cells at the four terminal erythroid differentiation stages: the proerythroblasts stage, the basophilic erythroblast stage, the polychromatic erythroblast stage and the orthochromatic erythroblast stage (Fig. 4D). AHSP expression was obviously increased during terminal erythroid differentiation from proerythroblasts to orthochromatic erythroblasts (Supplementary Fig. 3D). To exclude the possibility that tBHQ treatment promoted erythroid differentiation and hence increased AHSP expression levels in total Ter119+ cells, we cultured and treated isolated proerythroblasts and basophilic, polychromatic and orthochromatic erythroblasts with 20 μM tBHQ for 6 h. As shown in Fig. 4E, tBHQ treatment increased AHSP expression in erythroblasts at each individual stage (GCLM upregulation served as a biomarker for Nrf2 activation in mouse Ter119+ cells).

### 4.5 Nrf2 mediates the protective increase in AHSP in β-thalassemia

**Fig. 5:**
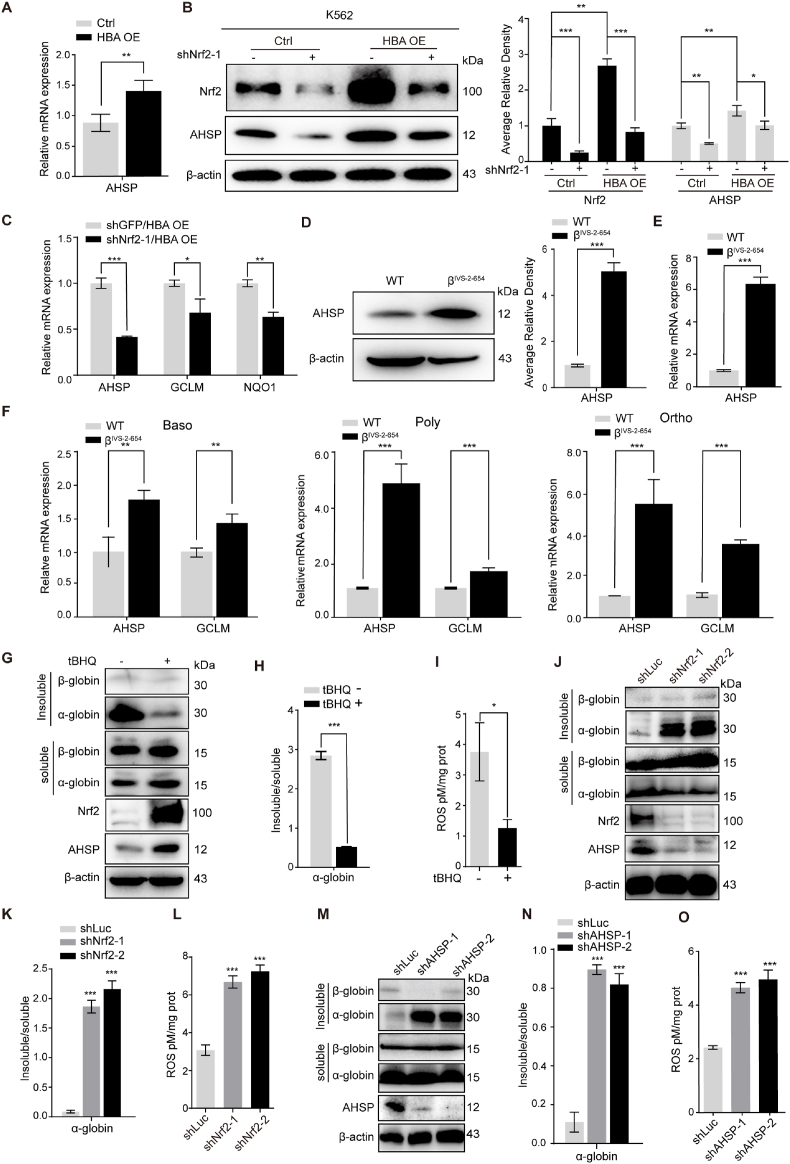
AHSP is upregulated in β-thalassemia models, and partially represses α-globin aggregation and ROS production. (A) Real-time PCR analysis of AHSP expression in α-globin-overloaded and control K562 cells. (the data are presented as the mean ± SD; **, *P* < 0.01; *n* = 3 *replicates*). (B) Representative Western blot analysis of Nrf2 and AHSP expression before and after Nrf2 knockdown in α-globin-overloaded and control K562 cells. AHSP and Nrf2 levels were assessed by densitometric quantification and normalized to β-actin levels (the data are presented as the mean ± SD; *, *P* < 0.005; **, *P* < 0.01; ***, *P* < 0.005; *n* = 4 *replicates*). (C) Real-time PCR analysis of AHSP, GCLM and NQO1 expression following Nrf2 knockdown in α-globin-overloaded K562 cells (the data are presented as the mean ± SD; *, *P* < 0.005; **, *P* < 0.01; ***, *P* < 0.005; *n* = 3 *replicates*). (D) Representative Western blot analysis of AHSP expression in splenic Ter119+ cells isolated from β^IVS-2-654^ thalassemic and wild-type mice. AHSP levels were assessed by densitometric quantification and normalized to β-actin levels (the data are presented as the mean ± SD; ***, *P* < 0.005; WT, *n* = 3 *mice;* β^IVS-2-654^, *n* = 3 *mice*). (E) Real-time PCR analysis of AHSP expression in splenic Ter119+ cells isolated from β^IVS-2-654^ thalassemic and wild-type mice. (the data are presented as the mean ± SD; ***, *P* < 0.005; WT, *n* = 3 *mice;* β^IVS-2-654^, *n* = 3 *mice*). (F) Real-time PCR analysis of AHSP and GCLM expression in bone marrow basophilic erythroblasts (Baso), polychromatic erythroblasts (Poly) and orthochromatic erythroblasts (Ortho) from wild-type and β^IVS-2-654^ thalassemic mice. (the data are presented as the mean ± SD; **, *P* < 0.01; ***, *P* < 0.005; *n* = 3 *replicates*). (G-I) β^IVS-2-654^ thalassemic mouse fetal liver (E14.5) derived erythroid cells were treated with tBHQ or DMSO during *in vitro* differentiation. (G) Representative Western blot analysis of insoluble and soluble α-/β-globin, Nrf2 and AHSP protein levels. (H) Ratio of insoluble to soluble α-globin (the data are presented as the mean ± SD; ***, *P* < 0.005; *n* = 3 *replicates*). (I) ROS detection assay (the data are presented as the mean ± SD; *, *P* < 0.05; n = 3 *replicates*). (J-O) RNA interference of Nrf2 (shNrf2) or AHSP (shAHSP) in β^IVS-2-654^ thalassemic mouse fetal liver (E14.5) derived erythroid cells. Control cells were transfected with shRNA targeting luciferase (shLuc). (J, M) Representative Western blot analysis of insoluble and soluble α-/β-globin, Nrf2 (for Panel J only) and AHSP protein levels. (K, N) Ratio of insoluble to soluble α-globin levels (the data are presented as the mean ± SD; ***, *P* < 0.005; *n* = 3 *replicates*). (L, O) ROS detection assay (the data are presented as the mean ± SD; ***, *P* < 0.005; n = 3 *replicates*).

We next examined whether AHSP expression was elevated in our cellular model of β-thalassemia, in which the Nrf2 antioxidant response signaling pathway was activated by endogenous ROS production. Real-time PCR analysis of α-globin-overloaded and control K562 cells showed increased AHSP expression following overexpression of free α-globin (Fig. 5A). To further investigate the role of Nrf2 in AHSP overexpression, we knocked down Nrf2 expression in α-globin-overloaded and control K562 cells. As shown in Fig. 5B, the protein levels of AHSP were decreased significantly in both control and α-globin-overloaded K562 cells following Nrf2 interference, suggesting that Nrf2 mediated AHSP upregulation upon α-globin overexpression. Nrf2 interference also inhibited AHSP mRNA expression in α-globin-overloaded K562 cells (GCLM and NQO1 upregulation served as biomarkers for Nrf2 activation in K562 cells) (Fig. 5C).

We further used the murine β^IVS-2-654^ model to determine whether AHSP expression is altered under genuine thalassemic conditions. Erythroid Ter119+ cells were magnetically isolated from the spleens of β^IVS-2-654^ mice and their WT littermates. Western blotting and real-time PCR revealed the significantly elevated expression of AHSP in splenic Ter119+ cells from β^IVS-2-654^ thalassemic mice (Fig. 5D and E). Because AHSP is stimulated during erythroid differentiation, we further sorted Ter119+ bone marrow cells into basophilic, polychromatic and orthochromatic erythroblasts using flow cytometry-based cell sorting (Supplementary Fig. 4). Real-time PCR analysis revealed that both AHSP and GCLM expression was increased in erythroblasts from β^IVS-2-654^ thalassemic mice at each stage compared with those from WT littermates (Fig. 5F). To examine the effects of AHSP overexpression on β-thalassemia pathology, we treated erythroid cells derived from E14.5 β^IVS-2-654^ thalassemic mouse fetal livers with tBHQ or infected them with retroviruses expressing shRNA targeting Nrf2 or AHSP. The thalassemic cells showed high insoluble α-globin and ROS levels, as expected (Fig. 5 G and I). Treatment of these cells with 20 μM tBHQ activated Nrf2 and AHSP expression, this activation was accompanied by an obviously reduced insoluble α-globin content (Fig. 5G, Supplementary Fig. 5 A), a decreased ratio of insoluble to soluble α-globin (Fig. 5H) and diminished ROS levels (Fig. 5I). In contrast, Nrf2 interference attenuated AHSP expression or AHSP knockdown in the cells, exacerbated α-globin precipitation (Fig. 5 J and M, Supplementary Fig. 5 B and C), increased the ratio of insoluble to soluble α-globin (Fig. 5K and N) and increased ROS levels (Fig. 5L and O). Flow cytometry analysis of CD71 (transferrin receptor) and Ter119 expression classified mouse fetal liver-derived erythroid cells into five groups (R1-R5) representing continuous stages in erythroblast differentiation. As shown in Supplementary Fig. 5 D-F, tBHQ treatment and Nrf2 or AHSP knockdown inducing mild changes in the R1 to R5 populations. Correspondingly, β-globin expression was not obviously changed with these treatments (Fig. 5G, J and 5 M). The results suggest that the observed modifications to insoluble α-globin content associated with tBHQ treatment and Nrf2 or AHSP knockdown described above were mainly due to the Nrf2-AHSP-mediated antioxidant response.

### 4.6 Nrf2 and small Mafs bind to and transactivate the MARE-1 element downstream of the AHSP TSS

**Fig. 6:**
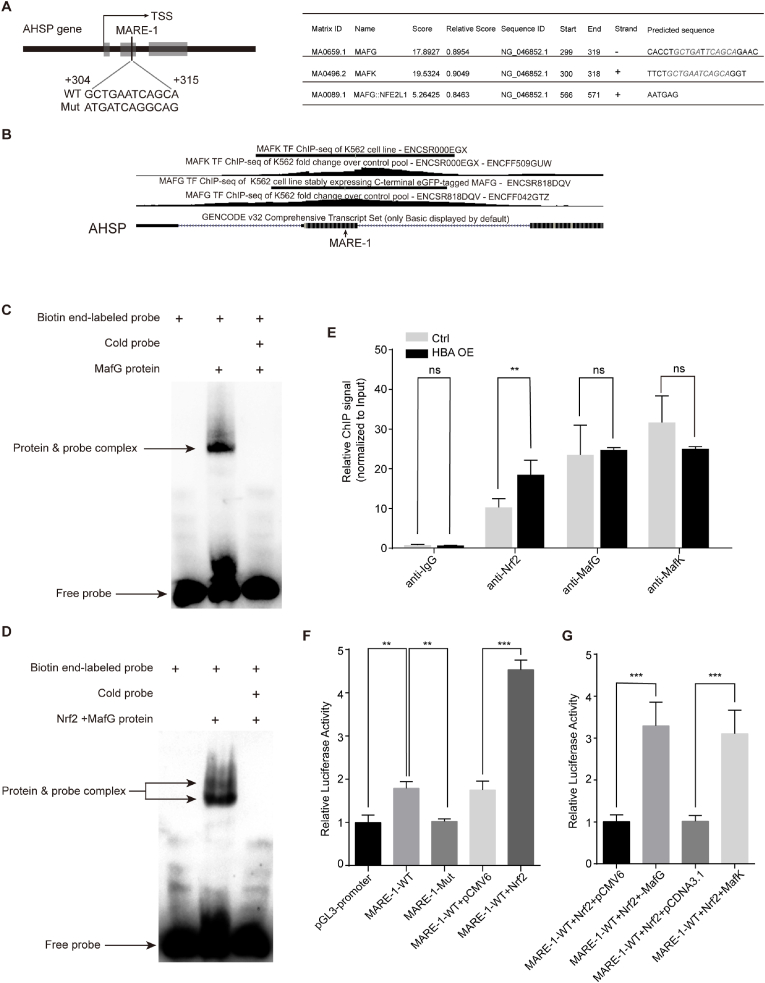
Nrf2 and MafG occupy and transactivate the MARE-1 element at proximal promoter region of AHSP. (A) JASPAR analysis predicted a putative MafG/K binding site, MARE-1, located +304 to +315 bp downstream of the AHSP transcriptional initiation site. Sequences of the original MARE-1 and the mutant used in (F) are shown. (B) ChIP-seq data showing MafG and MafK binding status in the proximal promoter region of AHSP in K562 cells (data obtained from MafG: GSE92076 and MafK: GSE31477). (C and D) EMSA of the MARE-1. A probe containing the MARE-1 was incubated with purified MafG protein (C) or purified Nrf2 and MafG proteins (D). Excess unlabeled cold probe (200-fold) was included as indicated. (E) ChIP analysis of Nrf2, MafG and MafK occupancy at the MARE-1 site on the AHSP gene in α-globin-overloaded and control K562 cells (the data are presented as the mean ± SD; ns, Not significant; **, *P* < 0.01; *n* = 3 *replicates*). (F) Dual-luciferase reporter assays to determine the enhancer activity of the MARE-1-WT fragment (+52 to +552 bp from the AHSP transcriptional initiation site), the effect of the MARE-1 element mutation, and the ability of Nrf2 to transactivate the enhancer activity of the MARE-1-WT fragment. The pGL3-promoter vector with the SV40 promoter was used as a control (the data are presented as the mean ± SD; **, *P* < 0.01; ***, *P* < 0.005; *n* = 3 *replicates*). (G) Dual-luciferase reporter assays to determine the ability of MafK or MafG to cooperate with Nrf2 in transactivating the enhancer activity of the MARE-1-WT fragment (the data are presented as the mean ± SD; ns, ***, *P* < 0.005; *n* = 3 *replicates*).

CNC family proteins recognize MARE/ARE sites primarily by forming heterodimers with small Mafs (sMafs)[30, 31]. MafG and MafK are the two primary erythroid sMafs [32]. To elucidate the molecular mechanisms underlying thalassemic AHSP overexpression, we investigated Nrf2 and sMafs occupancy around the AHSP gene. The sMaf motif MARE-1 at +304∼+315 bp downstream of the transcription start site (TSS) was identified by JASPAR analysis [33] (Fig. 6A). Further bioinformatics analysis of sMaf ChIP-Seq data in K562 cells indicated that the MARE-1 site was highly enriched for binding of MafG and enriched to a lesser degree for binding of MafK (Fig. 6B). We evaluated the ability of this element to bind MafG and Nrf2 by electromobility shift assay (EMSA). The MARE-1 probe and purified MafG protein (Fig. 6C) or the MafG and Nrf2 proteins in combination (Fig. 6D) produced shifted protein-probe complex bands. The complex was completely outcompeted by an excess of cold probe. We also performed EMSA analysis using the MARE-1 probe and purified MafK protein (Supplementary Fig. 6A) or both the MafK and Nrf2 proteins (Supplementary Fig. 6B), but the shifted bands were much weaker. Chromatin immunoprecipitation (ChIP) analysis of α-globin-overloaded and control K562 cells showed that Nrf2, MafG and MafK all occupied the MARE-1 site surrounding the region (Fig. 6E) but not a negative control region downstream of the AHSP gene (Supplementary Figs. 6C–D). Notably, α-globin overload specifically induced the binding of Nrf2 to MARE-1 (Fig. 6E). Wild-type MARE-1 (MARE-1-WT) but not mutated MARE-1 (MARE-1-Mut) enhanced the transcriptional activity of the reporter gene when inserted between the *Nhe*I and *Xho*I sites of the pGL3 promoter, and Nrf2 expression increased the activity of MARE-1-WT in the reporter assay (Fig. 6F). In addition, cotransfection of Nrf2 and MafG or MafK further transactivated transcription of the MARE-1-WT reporter (Fig. 6G).

### 4.7 MafG cooperates with Nrf2 to upregulate AHSP expression

**Fig. 7:**
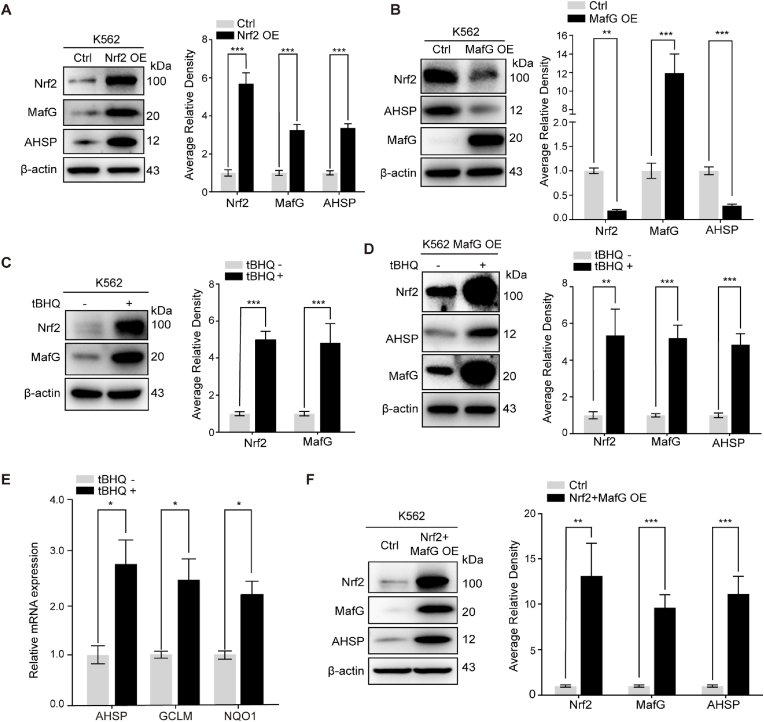
MafG facilitates Nrf2-mediated regulation of AHSP expression. (A) Representative Western blot analysis of Nrf2, MafG and AHSP expression in Nrf2-overexpressing and control K562 cells. MafG, AHSP and Nrf2 levels were assessed by densitometric quantification and normalized to β-actin levels (the data are presented as the mean ± SD; ***, *P* < 0.005; *n* = 4 *replicates*). (B) Representative Western blotting analysis of Nrf2, MafG and AHSP expression in MafG-overexpressing and control K562 cells. MafG, AHSP and Nrf2 levels were assessed by densitometric quantification and normalized to β-actin levels (the data are presented as the mean ± SD; **, *P* < 0.01; ***, *P* < 0.005; *n* = 5 *replicates*). (C) Representative Western blotting analysis of Nrf2 and MafG expression in K562 cells with or without tBHQ treatment for 6 h. Nrf2 and MafG levels were assessed by densitometric quantification and normalized to β-actin levels (the data are presented as the mean ± SD; ***, *P* < 0.005; *n* = 4 *replicates*). (D-E) Representative Western blotting analysis of Nrf2, MafG and AHSP expression (D) and Real-time PCR analysis of AHSP, GCLM and NQO1 expression (E) in MafG-overexpressing K562 cells with or without tBHQ treatment for 6 h (the data are presented as the mean ± SD; *, *P* < 0.05; **, *P* < 0.01; ***, *P* < 0.005; D: *n* = 3 *replicates;* E: *n* = 5 *replicates*). (F) Representative Western blot analysis of Nrf2, MafG and AHSP expression in Nrf2 and MafG double-overexpressing and control K562 cells. Nrf2, MafG and AHSP levels were assessed by densitometric quantification and normalized to β-actin levels (the data are presented as the mean ± SD; **, *P* < 0.01; ***, *P* < 0.005; *n* = 3 *replicates*).

To determine whether MafG facilitated Nrf2 to promote endogenous AHSP expression, we combined MafG overexpression with Nrf2 upregulation. Fig. 7A shows that Nrf2 overexpression in K562 cells enhanced MafG expression, and this enhancement was accompanied by elevated AHSP expression. Overexpression of MafG alone, however, decreased the expression of both Nrf2 and AHSP (Fig. 7B). No change in Nrf2 mRNA expression was detected (Supplementary Fig. 7), suggesting that differences in Nrf2 translation efficiency or protein degradation upon MafG overexpression may lead to a decrease in Nrf2 protein. Overexpression of MafG, together with a decrease in the Nrf2 level, may tend to cause formation of a repressive MafG homodimer rather than an active MafG/Nrf2 heterodimer and repress AHSP expression. Because tBHQ activates Nrf2, we then examined changes in Nrf2 and MafG protein levels in response to tBHQ. As shown in Fig. 7C, both Nrf2 and MafG were activated by tBHQ in K562 cells. tBHQ treatment clearly increased AHSP expression in MafG-overexpressing K562 cells (Fig. 7D), and the effect was stronger than that observed for tBHQ treatment in control K562 cells (Fig. 2D). Real-time PCR analysis confirmed the activation of Nrf2 target genes (GCLM and NQO1) and AHSP at mRNA level in tBHQ-treated MafG-overexpressing K562 cells (Fig. 7E). Finally, we overexpressed Nrf2 and MafG simultaneously in K562 cells, and coexpression led to a much more obvious increase in AHSP expression than overexpression of Nrf2 alone (Fig. 7F).

## 5 Discussion

AHSP plays important roles in limiting ROS production associated with free α-globin and maintaining the erythroid redox balance both during normal erythropoiesis and in thalassemic cells [20,[34], [35], [36]]. In the present study, we found that AHSP is a target gene of the Nrf2-ARE signaling pathway. Using α-globin-overexpressing K562 cells and β^IVS-2-654^ thalassemic mice, we demonstrated that AHSP expression was elevated following excessive α-globin stimulation and partially protected thalassemic cells from α-globin precipitation. We further determined that the sMaf protein MafG is the primary binding partner for Nrf2 on the MARE-1 site for AHSP regulation (Graphical Abstract).

There have been scattered reports of AHSP overexpression in the erythroblasts of β^0^-thalassemia patients [37] and in HbE/β-thalassemia erythrocytes [38,39]. However, convincing evidence of the pathological overexpression of AHSP in thalassemia cells has proven elusive, perhaps because it is a secondary compensatory response [40] or due to the extensive genetic heterogeneity within this gene and its regulators. α-Globin-overexpressing K562 cells and knock-in mice containing the human β^IVS-2-654^ thalassemia gene, to different degrees, served as genetically unified models for studying the pathology of human β-thalassemia and the molecular regulation of AHSP. Exogenous α-globin in both soluble and insoluble forms and increased ROS levels support α-globin-overexpressing K562 cells as a suitable *in vitro* system to mimic β-thalassemia. In this cellular model, α-globin protein increased by 2.5-3-fold (soluble α-globin: 3.11-fold; insoluble α-globin: 2.63-fold, Fig. 1A) with unaltered β-globin levels, implying an α-/β-globin ratio of ∼2.5–3 (considering the largely equal amounts of α- and β-globin in control cells). This is comparable to that in the heterozygous β^IVS-2-654^ mice used in the study, which had a theoretical α-/β-globin ratio of ∼2. However, K562 cells represent erythroid progenitor cells with much lower globin expression than native erythroid cells. Thus, the free α-globin burden should be more severe in the thalassemia mouse model, which bears higher ROS levels (3.35 pM/mg protein in the mouse model versus 2.06 pM/mg protein in the cellular model) and represents an overt thalassemia phenotype. Our observations in both models supported feedback upregulation of AHSP under β-thalassemia condition.

We show in the present work that Nrf2 mediates AHSP overexpression, partially reverses α-globin aggregation and reduces ROS levels in thalassemic erythroid cells. In support of our observations, Wang et al. showed that transgenic introduction of AHSP to β^IVS-2-654^ thalassemic mice ameliorated thalassemia syndrome, improving red blood cell counts and hemoglobin levels and reducing anisocytosis in peripheral blood [41]. Note that two bands for insoluble alpha-globin were detected in western blotting of both thalassemia models (Figs. 1A and 5J), which might result from oxidative modifications like glutathionylation and/or heme-protein crosslink [42,43]. The elevated AHSP expression also raises the possibility of competition with β-globin, which may limit the formation of the HbA tetramer (α_2_β_2_). The binding affinity of ferrous α-globin for AHSP (*K*_*D*_ ≈ 17 nM) is much lower than that to β-globin (*K*_*D*_ ≈ 0.001 nM), making it easy for the latter to displace AHSP during HbA assembly. The transition of ferrous α-Globin/AHSP to a redox-inert ferric form, the major way for AHSP to protect α-globin, leads to an ∼100-fold increase in complex stability and kinetic slowdown but does not impede substitution by β-globin [19,35]. Moreover, a 10-fold increase in AHSP levels has not been found to interfere with HbA assembly, even under conditions of β-globin deficiency [44]. Our work, together with that of others, suggests that elevated AHSP can to some extent balance the deleterious effects of α-globin in β-thalassemia and does not seem to restrict HbA formation therein.

In addition to Nrf2, other CNC family members, such as p45NFE2 and Nrf1, are actively involved in maintaining the redox equilibrium of erythroid cells [[45], [46], [47]]. Other regulatory pathways, including those of heme-regulated inhibitor kinase (HRI) [48], iron-regulatory proteins (IRPs) [49], and the heme-binding protein Bach1 [50], are known to be either activated or suppressed in thalassemia cells. Previous work has suggested that the binding of IRP to the AHSP 3′-untranslated region (UTR) modulates AHSP mRNA stability in an iron concentration-dependent manner [51]. Therefore, it is likely that the combined effects of multiple signaling pathways are responsible for the overexpression of AHSP and its protective effects in thalassemia cells.

Small Maf proteins bind Nrf2 to target AREs and activate downstream genes. A previous study found by ChIP sequencing that MafG and Nrf2 often colocalized at regions around ARE motifs and proximal to cytoprotective genes [52]. In addition, the expression of MafG itself appears to be activated by Nrf2 [53]. In the present study, we observed significant Nrf2 and MafG occupancy at the MARE-1 downstream of the AHSP transcription start site, and Nrf2 binding was specifically enhanced in the α-globin-overexpressing cells. The selective binding of Nrf2/MafG to the MARE-1 site indicates that this element may be an effective new target for future population screens of single-nucleotide polymorphisms (SNPs) linking variations in AHSP expression to the severity of thalassemic pathology.

We analyzed several public gene expression datasets from Nrf2-activated/suppressed and control nonerythroid cells but found no significant changes in AHSP expression (Supplementary Tables 2–5[[54], [55], [56]]). Consistently, available ChIP-seq data for multiple nonerythroid cells revealed no or very weak Nrf2 and MafG binding at the MARE-1 site (Supplementary Fig. 9 A and B [57,58]). It is therefore likely that the regulatory effect of Nrf2 on AHSP expression represents an erythroid lineage-specific effect, but more studies will be necessary to confirm this possibility. Interestingly, enriched MafK signals at the MARE-1 site were found in lung A549 lung and HepG2 liver carcinoma cells (Supplementary Fig. 9C [57]), implying potential regulation of the AHSP gene by other CNC-bZIP family members.

## Declaration of competing interest

All authors disclosed no relevant relationships.
