# Supplementary material for: Nrf2 expands the intracellular pool of the chaperone AHSP in a cellular model of β-thalassemia
Source: Redox Biol. 2022 Jan 21;50:102239. doi: 10.1016/j.redox.2022.102239 (PMC8801382; doi:10.1016/j.redox.2022.102239)
Supplement: Multimedia component 1 [file mmc1.docx]

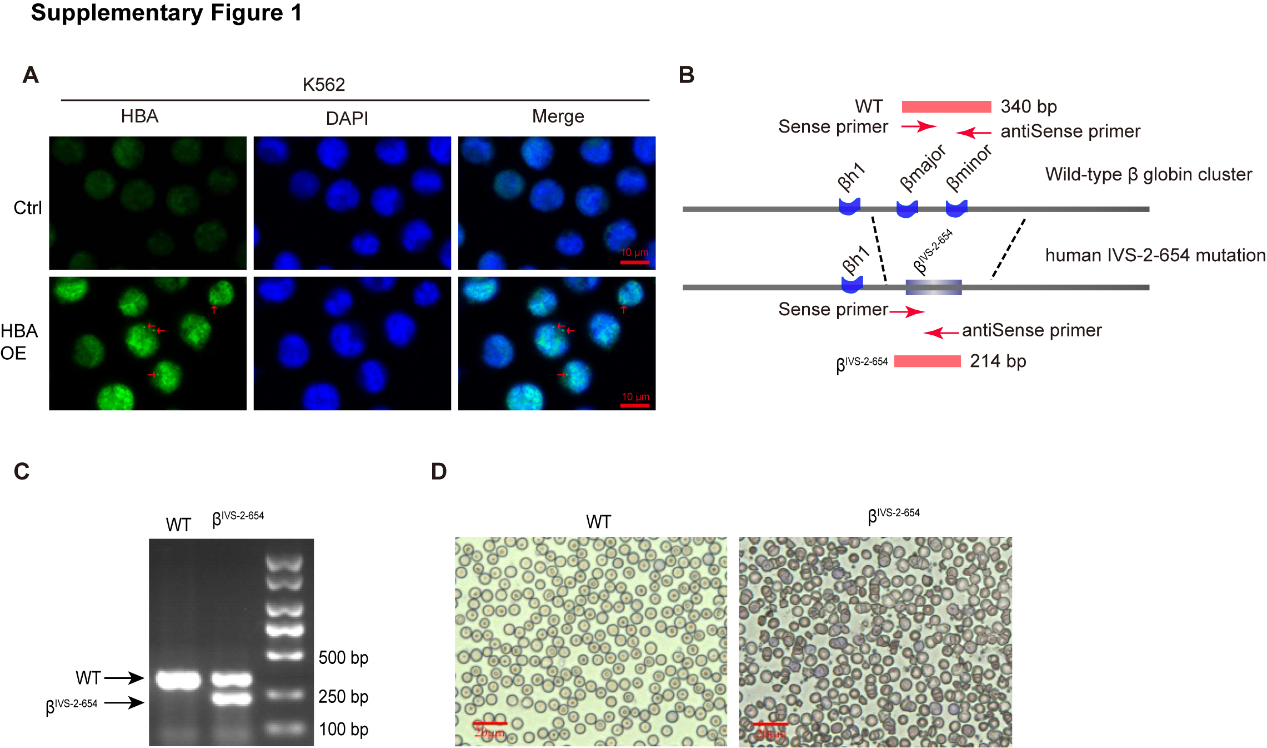


**Supplementary Figure 1. β-Thalassemia models.**

1. Immunofluorescence analysis showing the overexpression and aggregation (indicated by red arrows) of α-globin in α-globin-overexpressing K562 cells.
2. Genomic structure of β^IVS-2-654^ thalassemic mice carrying human β^IVS-2-654^-globin mutation and its wild-type control.
3. Genotyping of β^IVS-2-654^ thalassemic mice with primers designed to amplify the human β^IVS-2-654^-globin gene and mouse wild-type β-globin gene. The position of the PCR primers and the lengths of amplicons are indicated in (B).
4. Wright-Giemsa staining of blood smears from wild-type and β^IVS-2-654^ thalassemic mice.


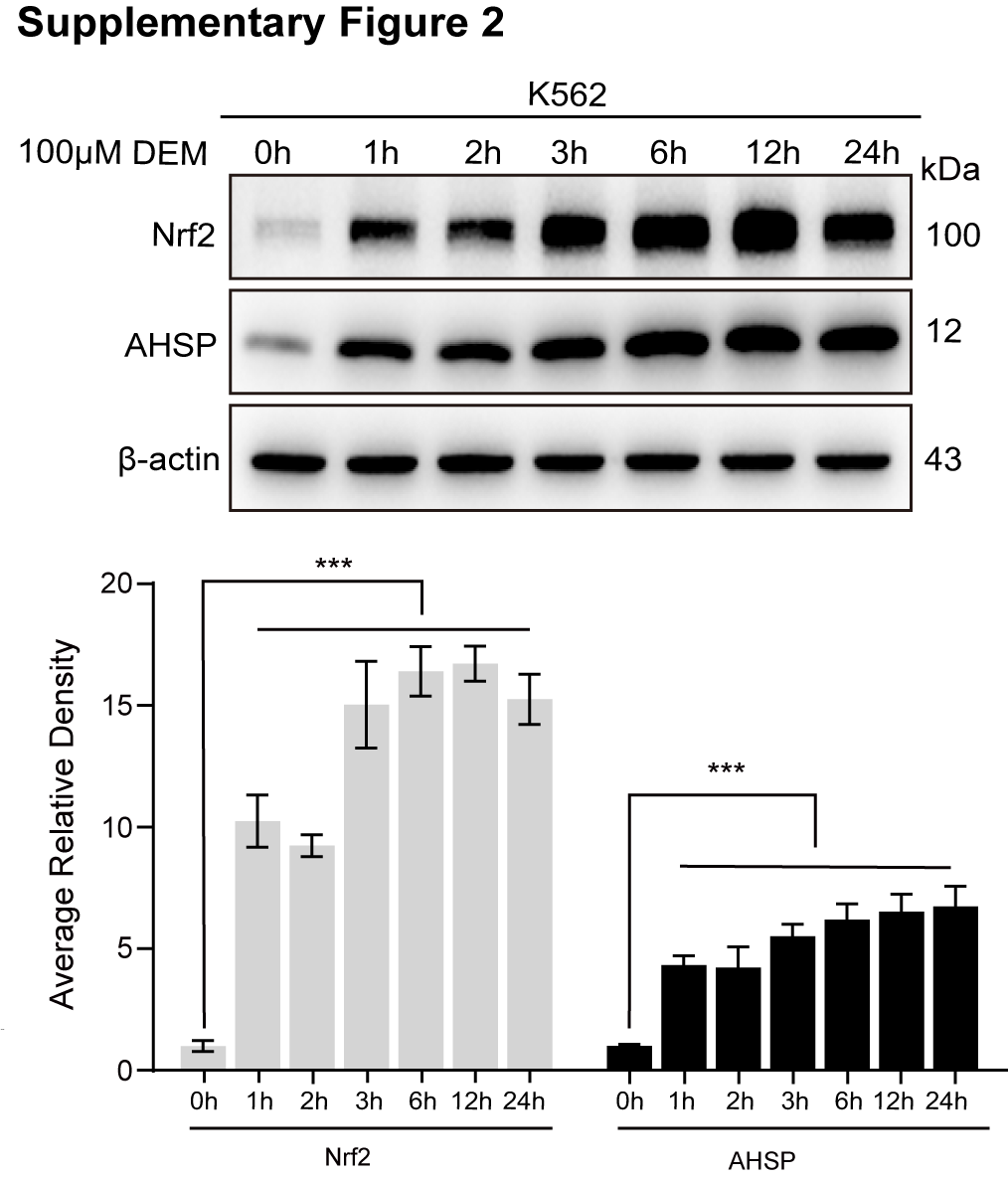


**Supplementary Figure 2. The Nrf2-specific activator DEM regulates the expression of AHSP.** Representative Western blot analysis of Nrf2 and AHSP expression activated by DEM. Nrf2 and AHSP levels were assessed by densitometric quantification and normalized to β-actin levels (the data are presented as the mean ± SD; ***, *P* <0.005; *n*=4 *replicates*).


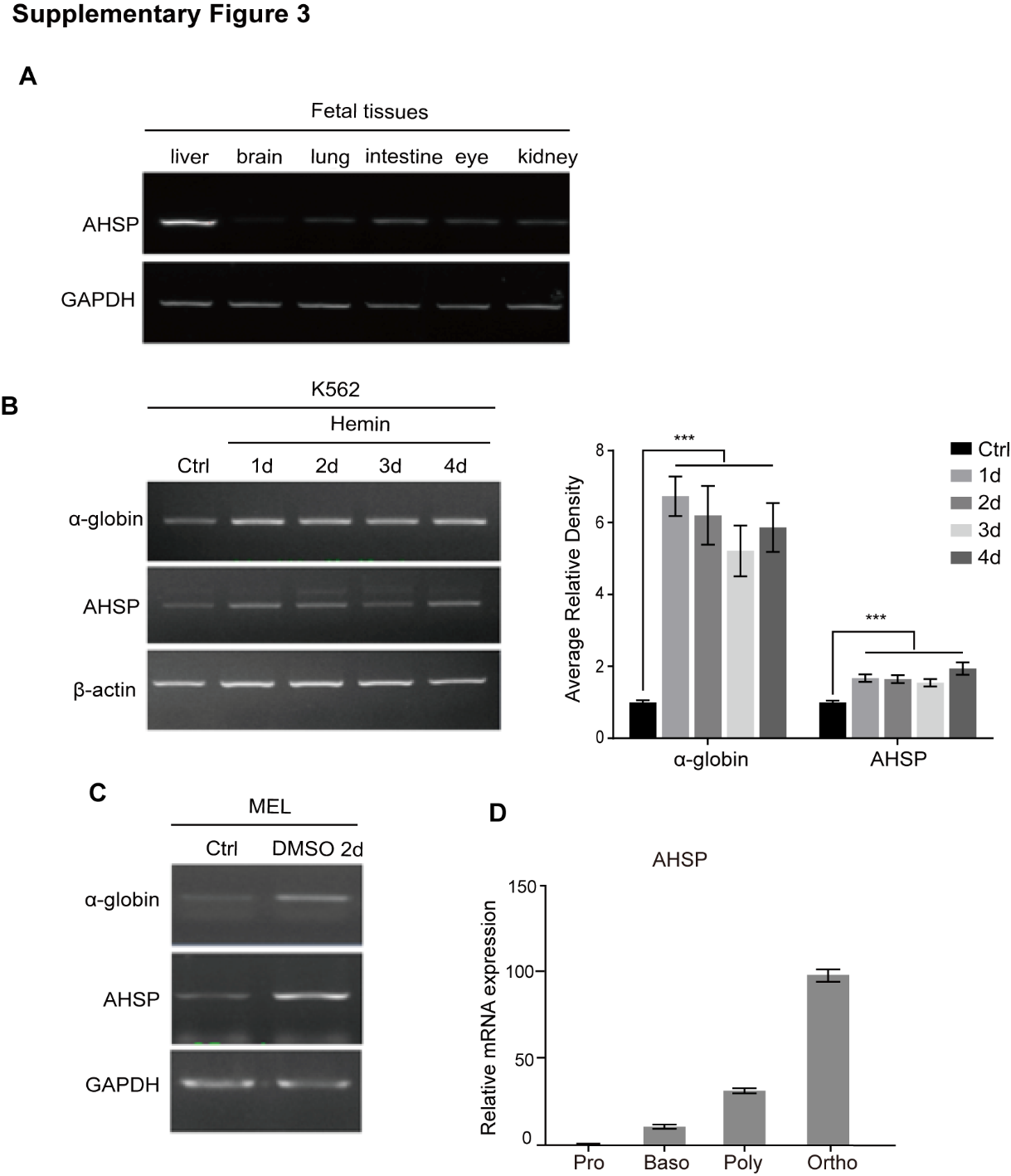


**Supplementary Figure 3. AHSP expression during erythroid differentiation.**

(A-C) RT-PCR analysis of the expression of AHSP in different fetal tissues (A), hemin-exposed K562 cells, in which α-globin and AHSP levels were assessed by densitometric quantification and normalized to β-actin levels (the data are presented as the mean ± SD; ***, *P* <0.005; *n*=3 *replicates*) (B), and DMSO-exposed MEL cells (C).

1. Real-time PCR analysis of the expression of AHSP in staged erythroblasts of wild-type mouse bone marrow cells (the data are presented as the mean ± SD; *n*=3 *replicates*).


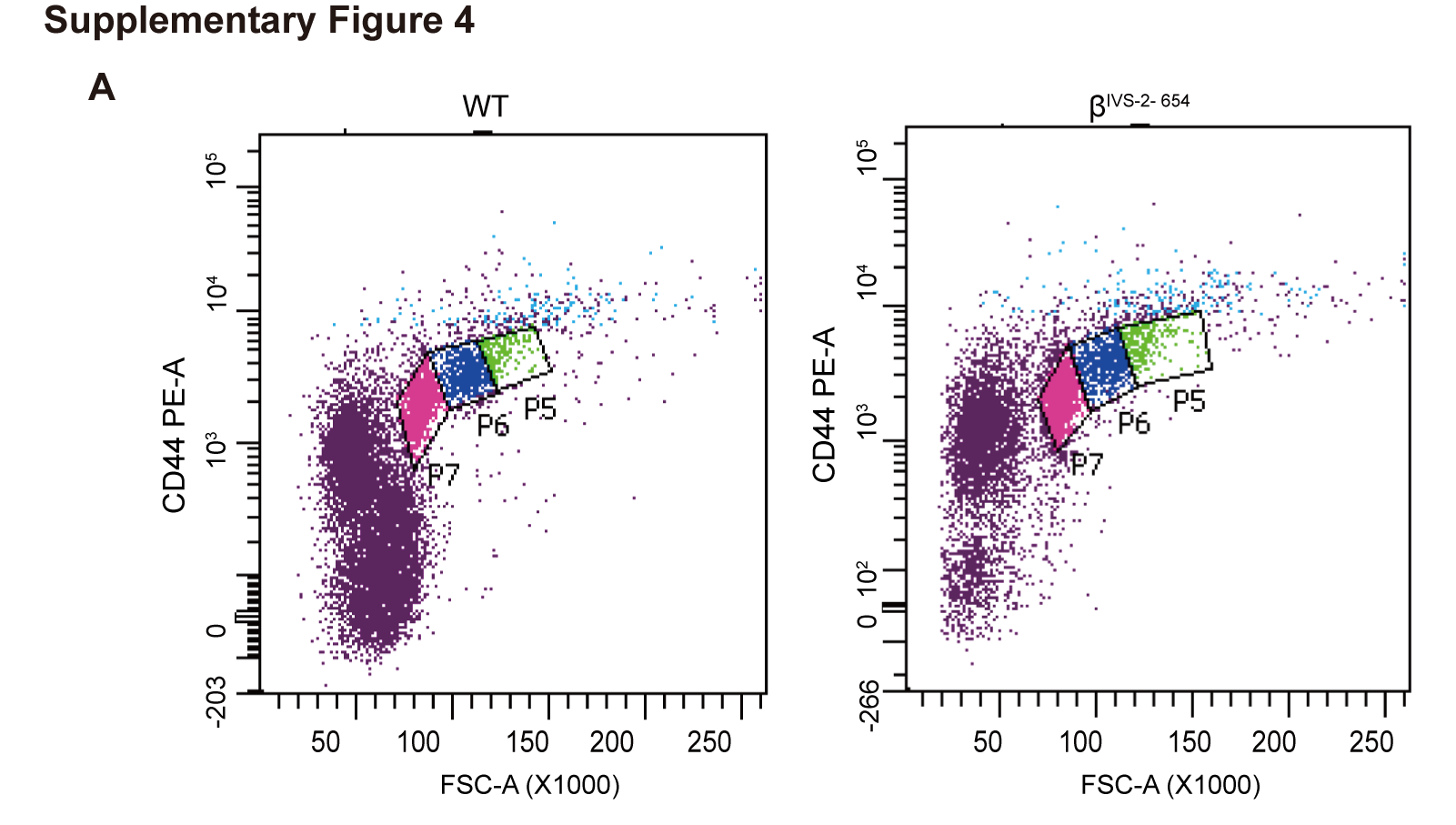


**Supplementary Figure 4. FACS isolation of staged erythroblasts from bone marrow cells of wild-type and β^IVS-2-654^ thalassemic mice.** Regions P5, P6 and P7 were basophilic erythroblasts (Baso), polychromatic erythroblasts (Poly) and orthochromatic erythroblasts (Ortho), respectively.


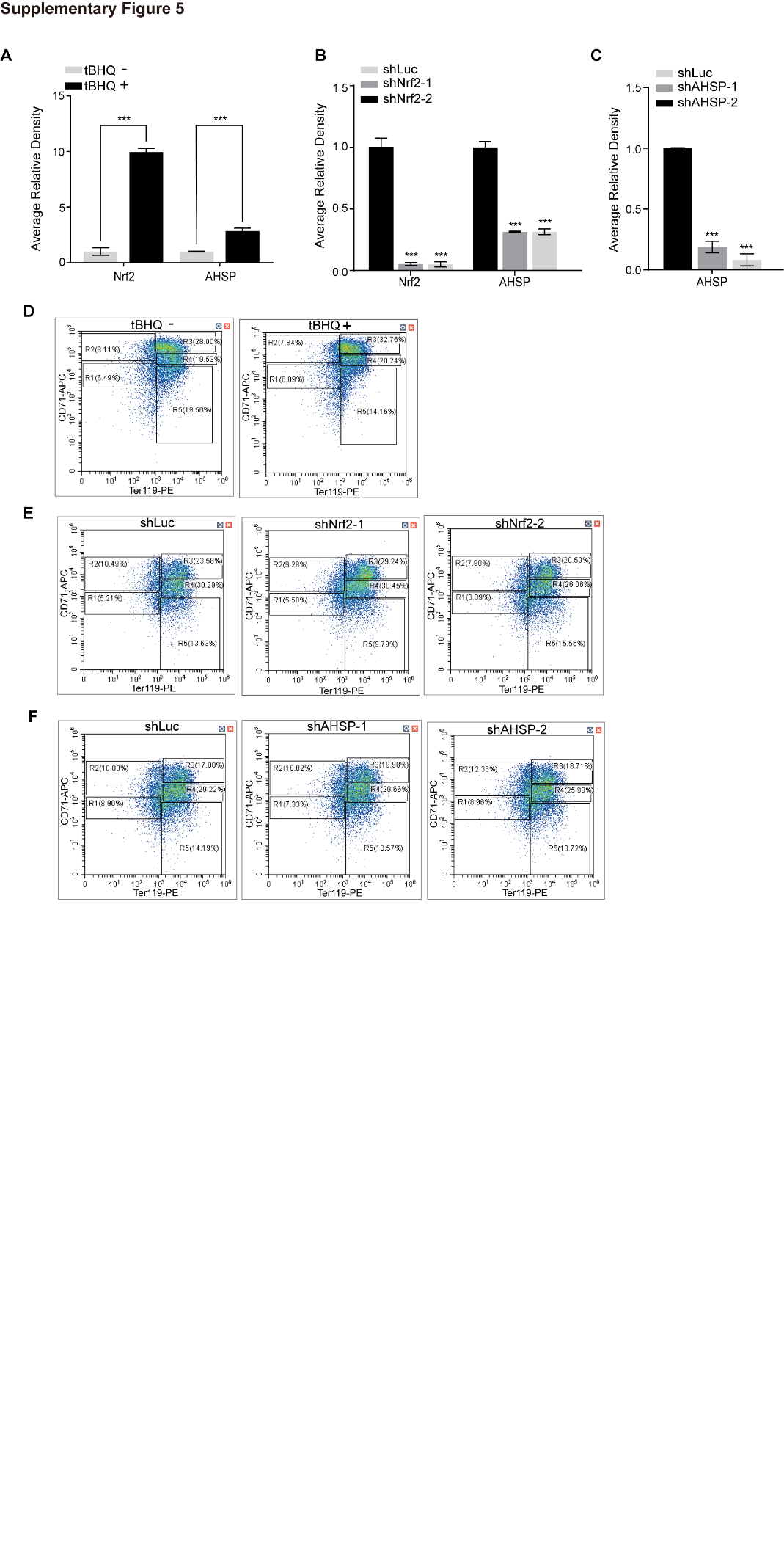


**Supplementary Figure 5. Quantification of Nrf2 and AHSP genes expression and cell differentiation in tBHQ-treated and Nrf2- or AHSP-knockdown erythroid cells derived from E14.5 β^IVS-2-654^ thalassemic mouse fetal livers.**

1. The Nrf2 and AHSP levels in Figure 5G were assessed by densitometric quantification and normalized to β-actin levels (the data are presented as the mean ± SD; ***, *P* <0.005; *n*=3 *replicates*).
2. The Nrf2 and AHSP levels in Figure 5J were assessed by densitometric quantification and normalized to β-actin levels (the data are presented as the mean ± SD; ***, *P* <0.005; *n*=3 *replicates*).
3. The AHSP levels in Figure 5M were assessed by densitometric quantification and normalized to β-actin levels (the data are presented as the mean ± SD; ***, *P* <0.005; *n*=3 *replicates*).

(D-F) Flow cytometry analysis of β^IVS-2-654^ thalassemic mouse fetal liver (E14.5) derived erythroid cells treated with DMSO or tBHQ (D), infected with retroviruses expressing shRNA targeting luciferase (shLuc) or Nrf2 (shNrf2) (E), or infected with retroviruses expressing shRNA targeting luciferase (shLuc) or AHSP (shAHSP) (F). Gates R1-R5 are erythroid progenitor cells and proerythroblasts (R1), proerythroblasts and early basophilic erythroblasts (R2), early and late basophilic erythroblasts (R3), polychromatophilic and orthochromatophilic erythroblasts (R4), and late orthochromatophilic erythroblasts and reticulocytes (R5), respectively.

**
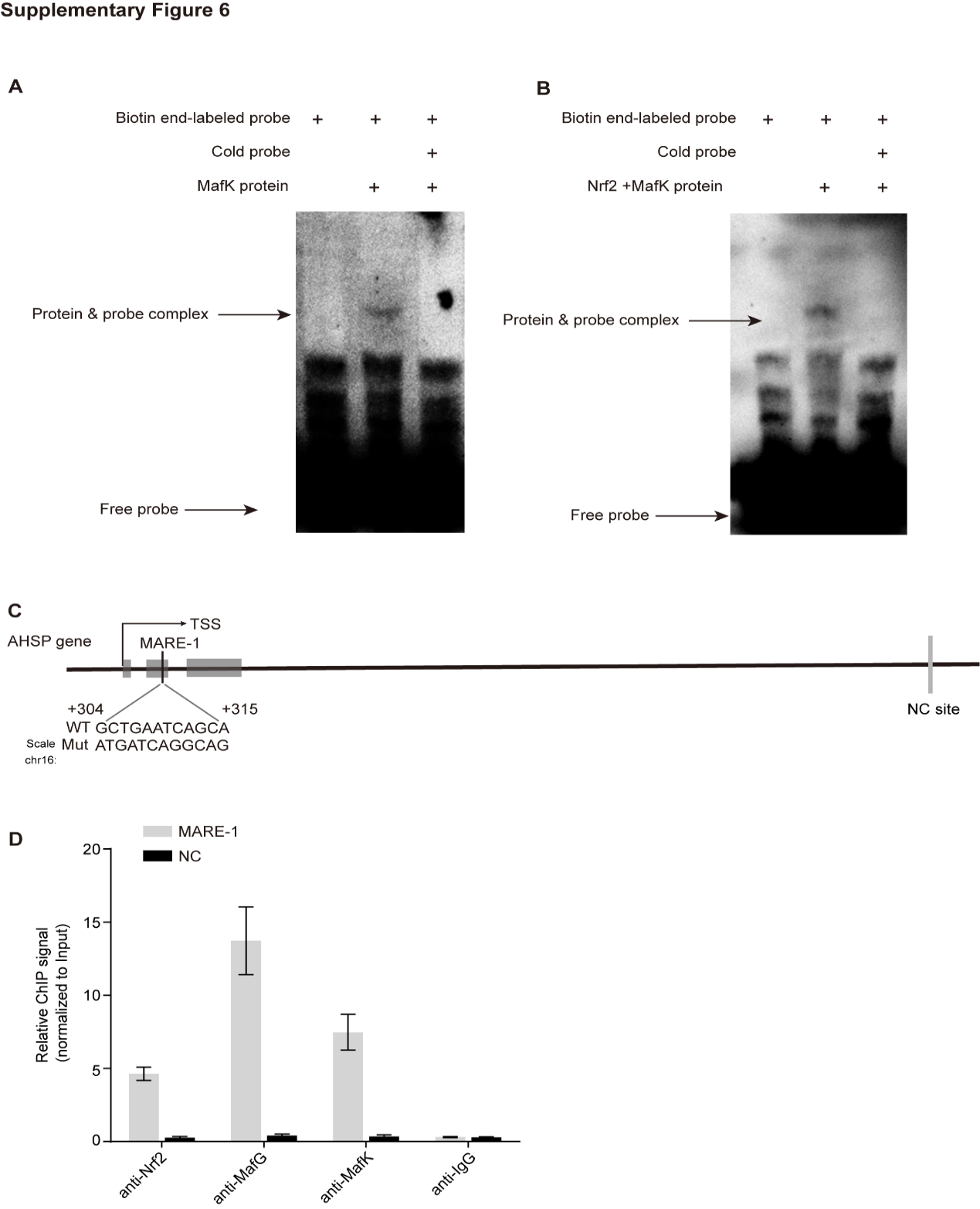
**

**Supplementary Figure 6.** **Nrf2 and sMaf occupancy of the proximal promoter region of AHSP.**

(A-B) EMSA of a probe containing the MARE-1 incubated with purified MafK protein (A) or purified Nrf2 and MafK proteins (B). Excess unlabeled cold probe (200-fold) was included as indicated.

(C) Schematic diagram showing the location of the MARE-1 site and negative control (NC) site used in (D).

1. ChIP analysis of Nrf2, MafG and MafK occupancy at the MARE-1 site and a downstream NC site in α-globin-overexpressing K562 cells.


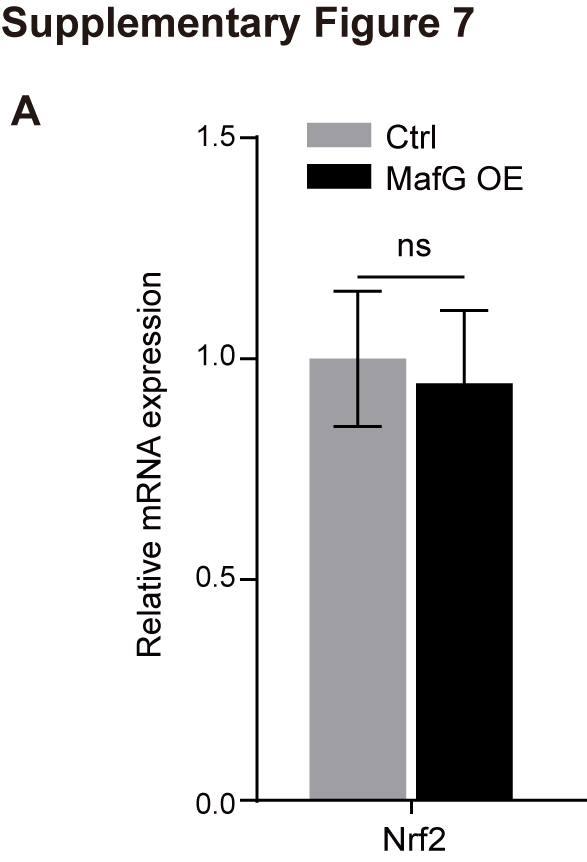


**Supplementary Figure 7. Real-time** **PCR analysis of Nrf2 mRNA expression in MafG-overexpressing and control K562 cells** (the data are presented as the mean ± SD; ns, not significant; n=3 replicates).

**
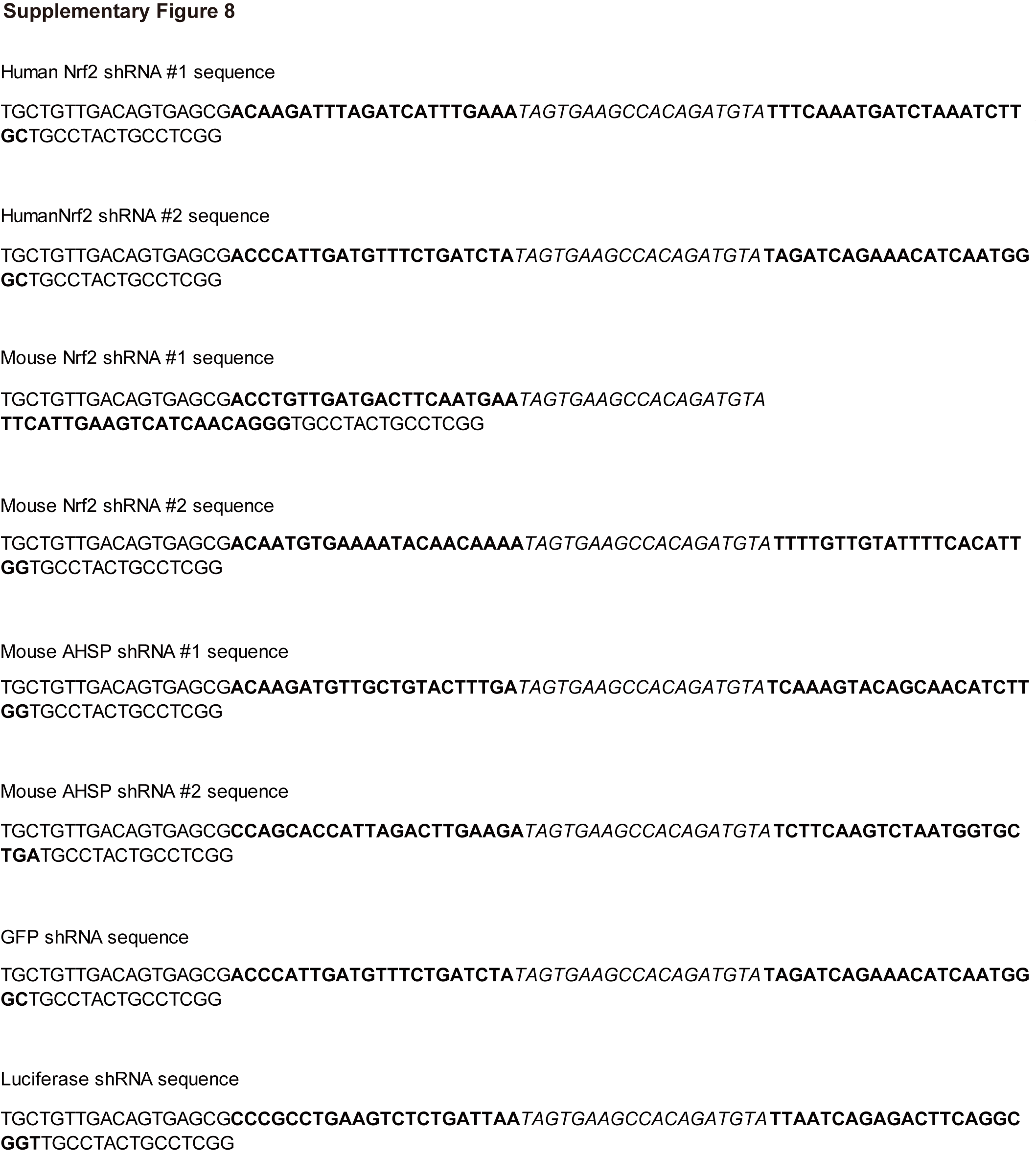
**

**Supplementary Figure 8.** **Sequences of shRNAs.** The sense and antisense strands are indicated in bold; the loops are indicated in italics.


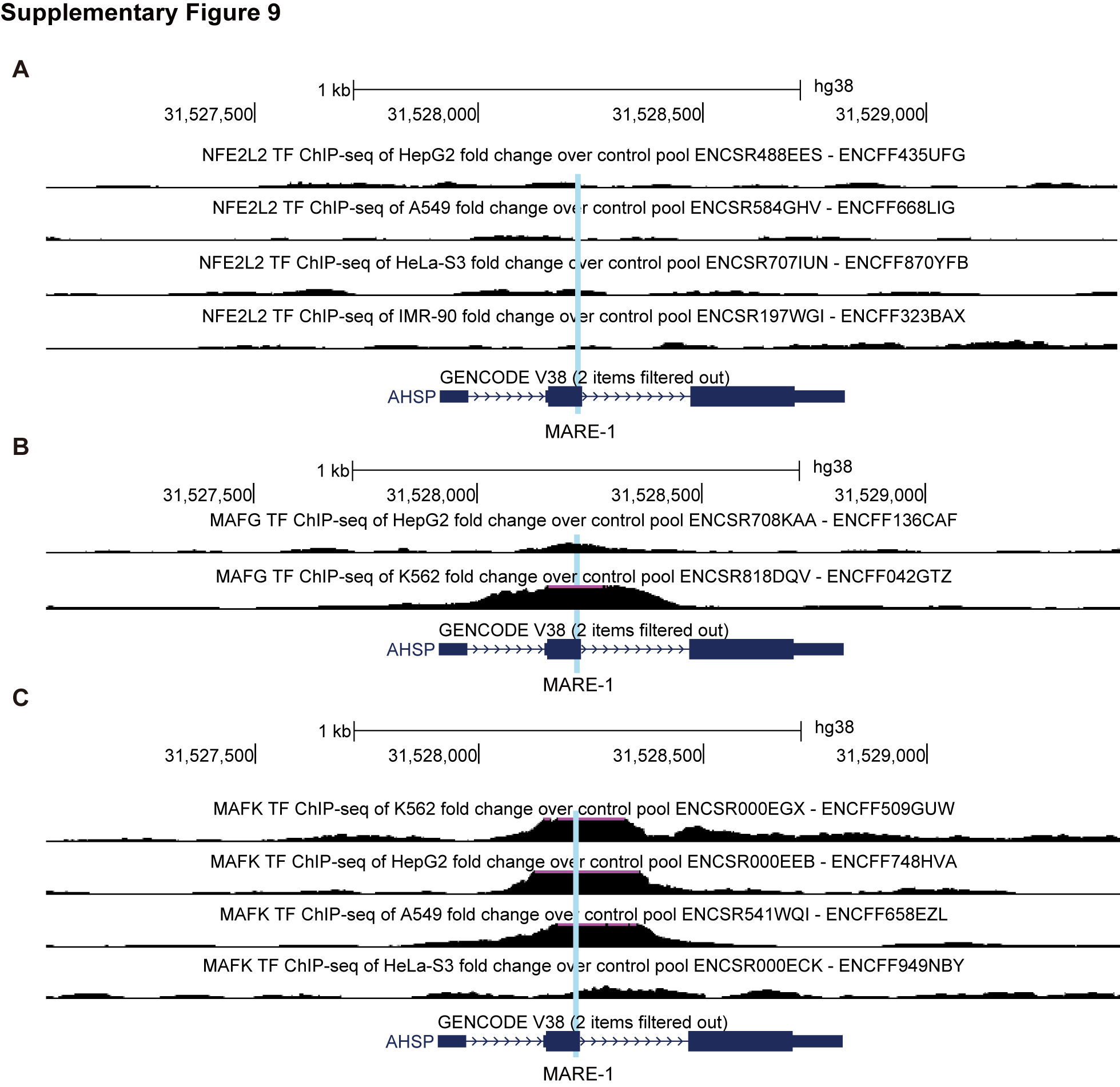


**Supplementary Figure 9. Nrf2 and sMaf binding status at the MARE-1 site in nonerythroid cell lines.** ChIP-seq data showing (A) the Nrf2 binding status at MARE-1 site in HepG2 (GSE91809, [1]), A549 (GSE91894, [1]), HeLa-S3 (GSE91997, [1]) and IMR-90 (GSE19565, [2]) cells; (B) the MafG binding status at the MARE-1 site in HepG2 (GSE169821, [1]) and K562 (GSE92076, [1]) cells (as an erythroid control); and (C) the MafK binding status at the MARE-1 site in K562 (as an erythroid control), HepG2, A549 and HeLa-S3 cells (GSE31477, [1]).

**
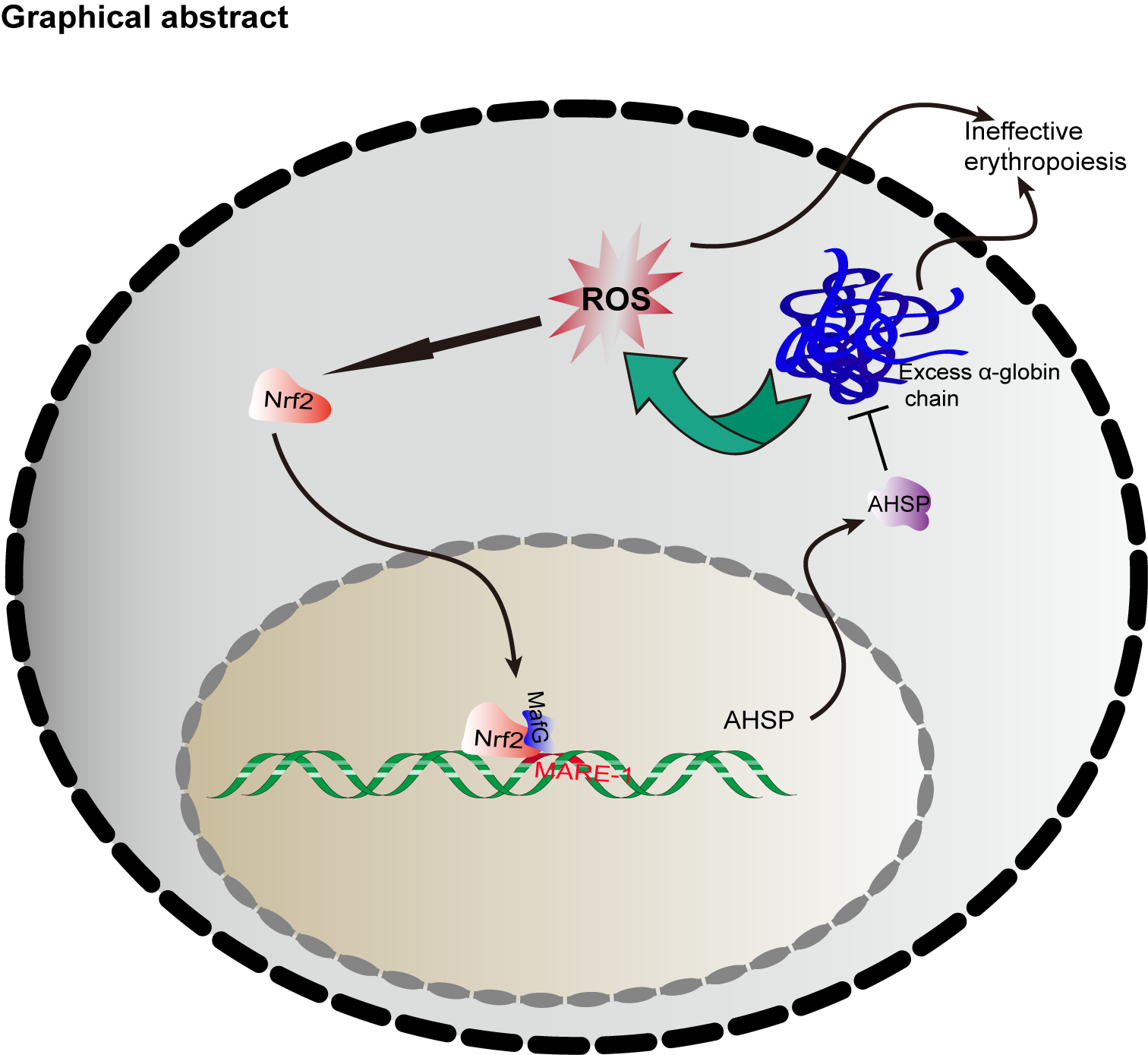
**

**Graphical Abstract. A model of ROS/Nrf2-mediated AHSP overexpression in β-thalassemia cells.** Excess α-globin chains catalyse the production of ROS in β-thalassemia cells, which causes ineffective erythropoiesis. Meanwhile, ROS stimulate the translocation of Nrf2 into the nucleus. Once there, Nrf2 cooperates with MafG to bind on MARE-1 site to promote AHSP expression. In turn, the expanded pool of AHSP proteins stabilizes the excess α-globin chains.

**
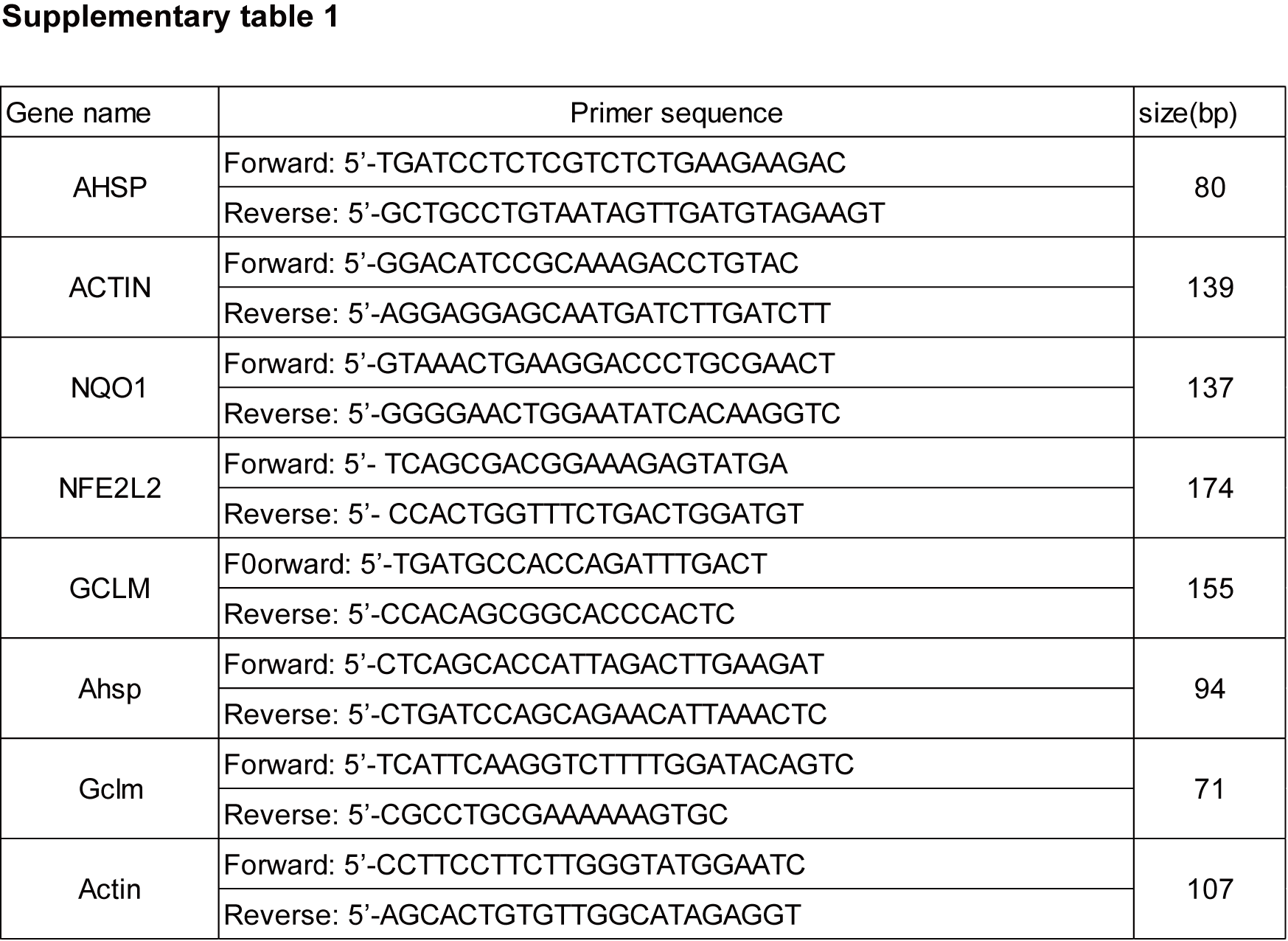
Supplementary Table 1.** **Real-Time RT- PCR primers**

**
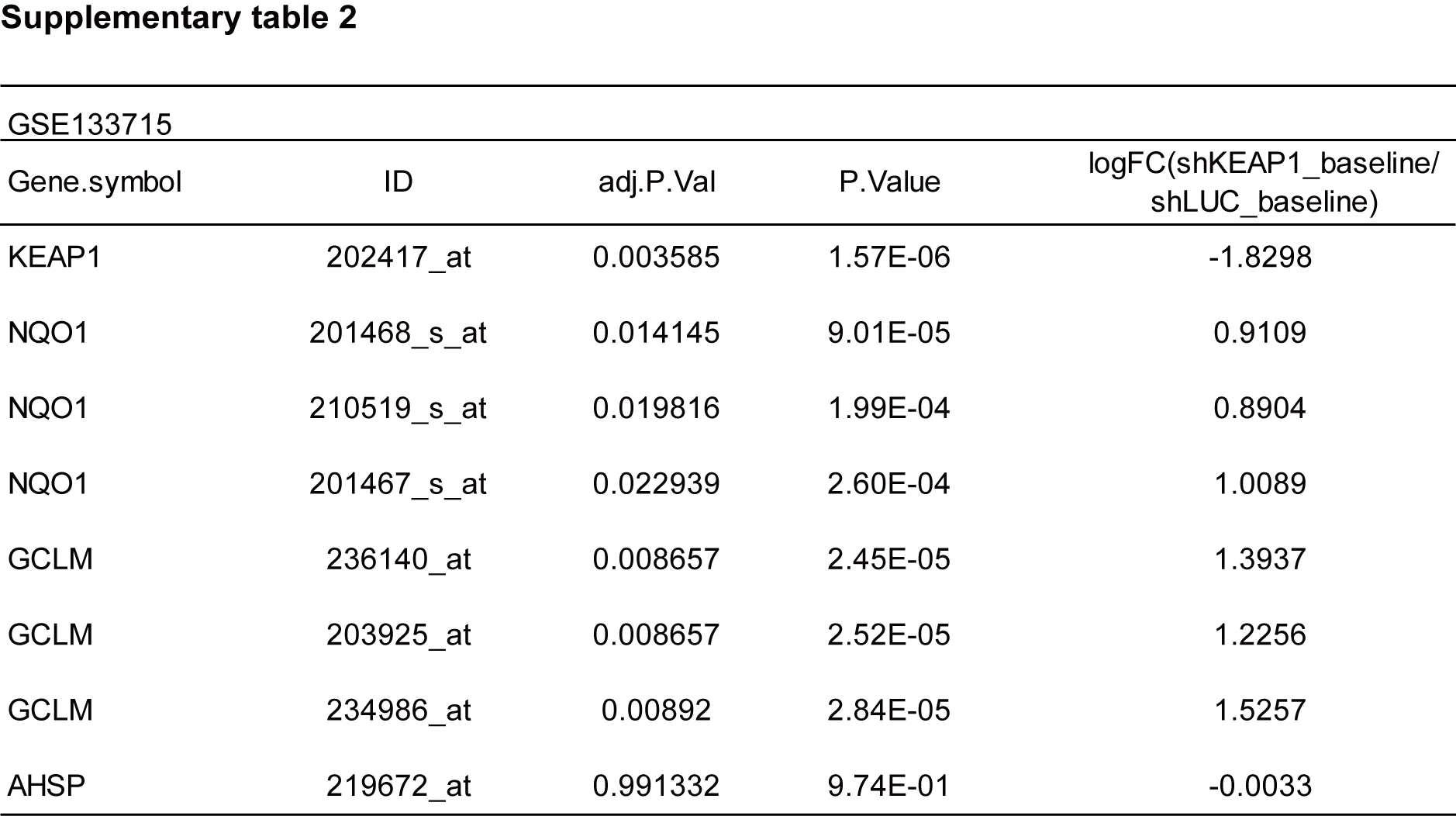
**

**Supplementary Table 2. Nrf2 activation does not upregulate AHSP expression in HCC515 cells under baseline stress conditions.** Expression changes of the KEAP1, NQO1, GCLM and AHSP genes based on microarray data of HCC515 lung cells with Dox-inducible KEAP1 knockdown under baseline stress conditions (GSE133715, [3]). The gene symbols and accession numbers are shown together with the corresponding *P* values adjusted for multiple testing (adj. P.Val), the *P* values (P.Value) for differential expression, and the log_2_ fold-change values between KEAP1-knockdown (shKEAP1) and control (shLUC) cells.

**
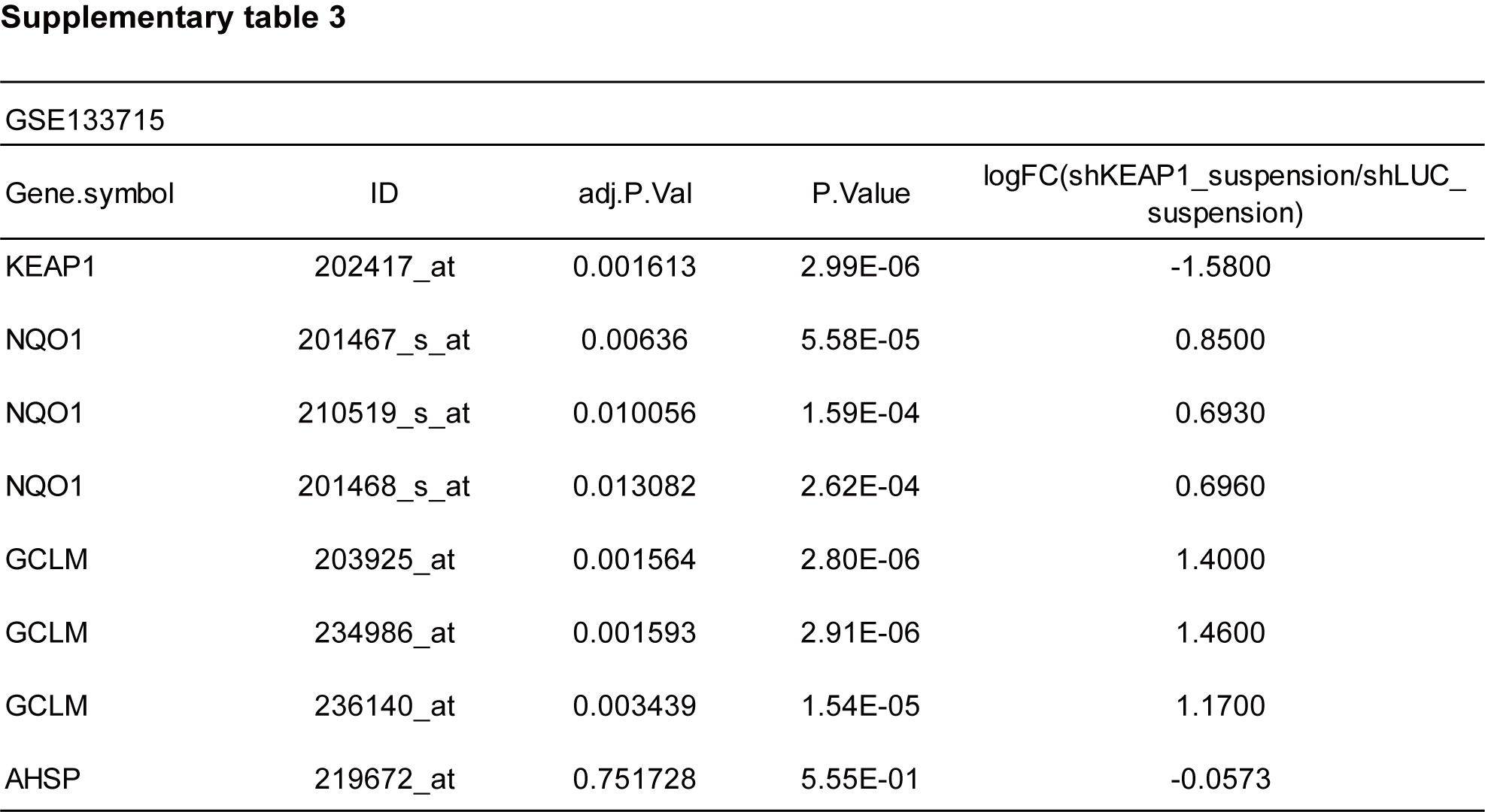
 Supplementary Table 3. Nrf2 activation does not upregulate AHSP expression in HCC515 cells under suspension stress conditions.** Expression changes of the KEAP1, NQO1, GCLM and AHSP genes based on microarray data of HCC515 lung cells with Dox-inducible KEAP1 knockdown under suspension stress conditions (GSE133715, [3]). The gene symbols and accession numbers are shown together with the corresponding *P* values adjusted for multiple testing (adj. P.Val), the *P* values (P.Value) for differential expression and the log_2_ fold-change values between KEAP1-knockdown (shKEAP1) and control (shLUC) cells.


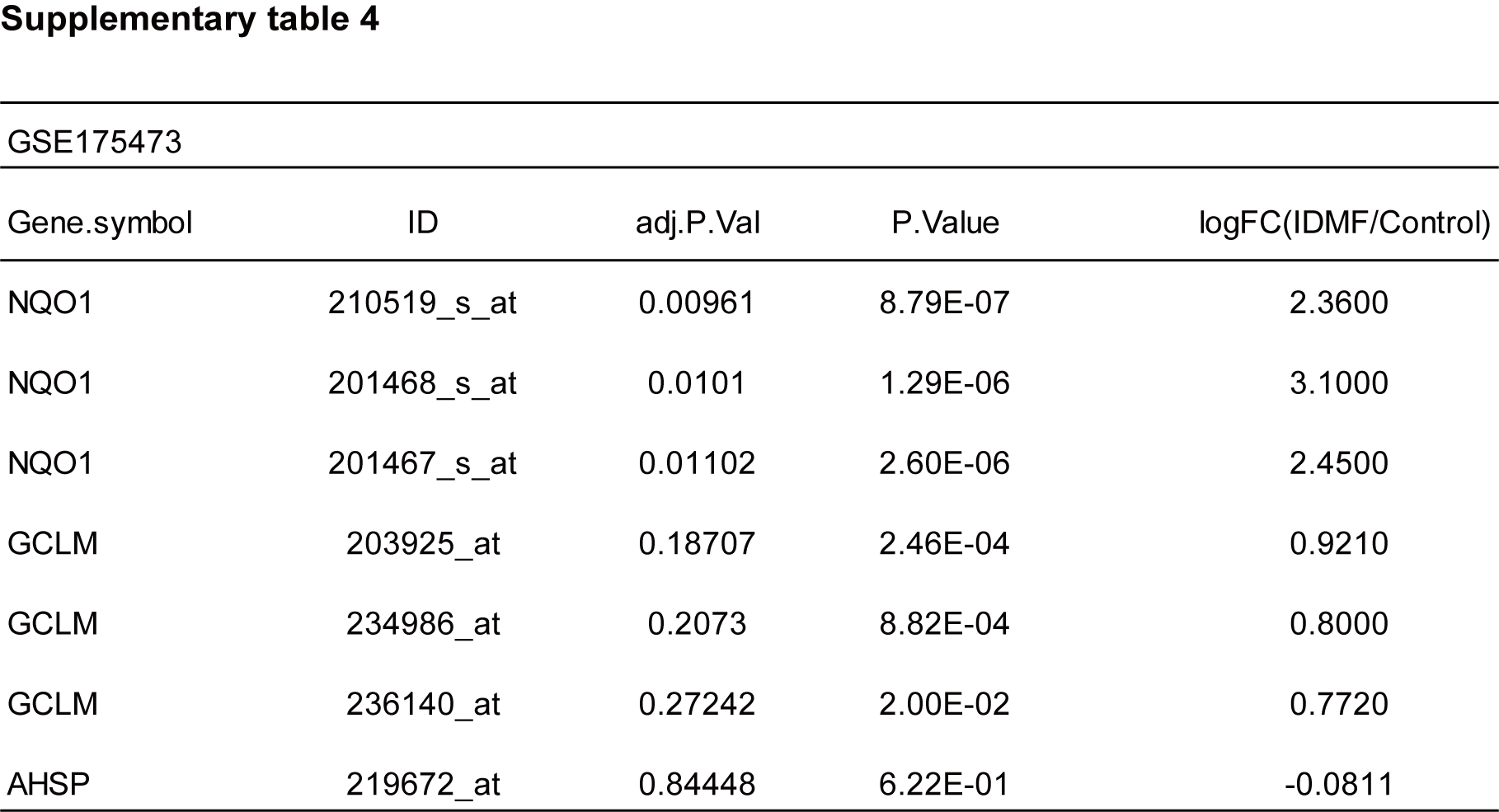


**Supplementary Table 4. IDMF activates Nrf2 but not AHSP expression in keratinocytes.** Expression changes of the NQO1, GCLM and AHSP genes based on microarray data of IDMF activated keratinocytes (GSE175473, [4]). The gene symbols and accession numbers are shown together with the corresponding *P* values adjusted for multiple testing (adj. P.Val), the *P* values (P.Value) for differential expression, and the log_2_ fold-change values between IDMF- and control (water)-treated cytokine-stimulated keratinocytes.


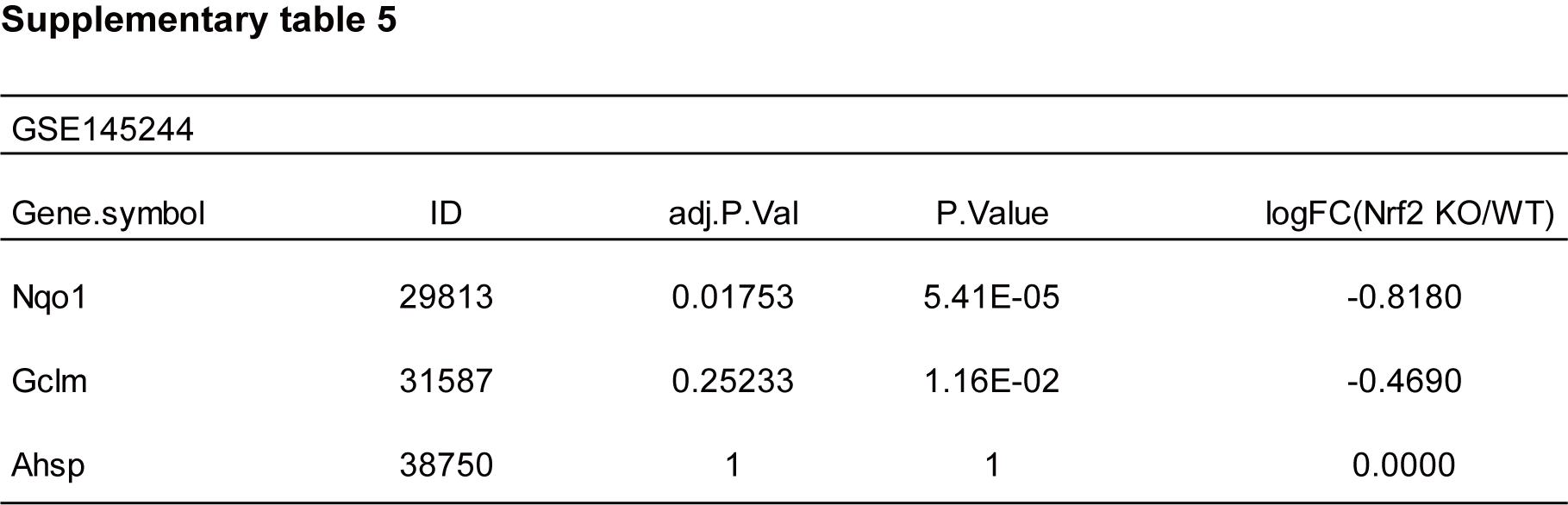


**Supplementary Table 5. Nrf2 does not mediate AHSP expression in mouse bone marrow-derived macrophages (BMDMs).** Expression changes of the NQO1, GCLM and AHSP genes based on microarray data of heme- and LPS- stimulated mouse BMDMs with Nrf2-KO vs. Nrf2-WT (GSE145244, [5]). The gene symbols and accession numbers are shown together with the corresponding *P* values adjusted for multiple testing (adj. P.Val), the *P* values (P.Value) for differential expression, and the log_2_ fold-change values between stimulated BMDMs from Nrf2-KO and WT mice.

**Reference**

[1] ENCODE Project Consortium, An integrated encyclopedia of DNA elements in the human genome, Nature. 489 (2012) 57-74. https://doi.org/10.1038/nature11247.

[2] E. Epp, A. Walther, G. Lepine, Z. Leon, Forward genetics in Candida albicans that reveals the Arp2/3 complex is required for hyphal formation, but not endocytosis, Mol Microbiol. 75 (2010) 1182-1198. https://doi.org/10.1111/j.1365-2958.2009.07038.x.

[3] A. Singh, A. Daemen, D. Nickles, S. M. Jeon, NRF2 Activation Promotes Aggressive Lung Cancer and Associates with Poor Clinical Outcomes, Clin Cancer Res. 27 (2021) 877-888. https://doi.org/10.1158/1078-0432.CCR-20-1985.

[4] Krzysztof Bojanowski, Collins U. Ibeji, Parvesh Singh, William R. Swindell, Ratan K. Chaudhuri, A Sensitization-Free Dimethyl Fumarate Prodrug, Isosorbide Di-(Methyl Fumarate), Provides a Topical Treatment Candidate for Psoriasis, JID Innovations,Volume 1, Issue 4, 2021, 100040, ISSN 2667-0267, https://doi.org/10.1016/j.xjidi.2021.100040.

[5] M. Pfefferle, G. Ingoglia, C. A. Schaer, A. Yalamanoglu, Hemolysis transforms liver macrophages into antiinflammatory erythrophagocytes, J Clin Invest. 130 (2020) 5576-5590. https://doi.org/10.1172/JCI137282.
